# Association of skill and errors with outcomes in robotic rectal cancer surgery

**DOI:** 10.1007/s00464-025-12393-x

**Published:** 2025-12-10

**Authors:** M. Boal, C. Reali, R. Duhoky, T. Gill, J. Khan, D. Miskovic, N. Francis

**Affiliations:** 1 The Griffin Institute, Northwick Park & St Marks’ Hospital, London, UK; 2 Wellcome/ESPRC Centre for Interventional Surgical Sciences (WEISS), University College London (UCL), London, UK; 3 Yeovil District Hospital, Somerset Foundation NHS Trust, Yeovil, UK; 4 Queen Alexandra Hospital, Portsmouth Hospitals University NHS Trust, Portsmouth, UK; 5 North Tees and Hartlepool NHS Foundation Trust, Stockton-on-Tees, UK; 6 St Marks National Bowel Hospital, London North West University Healthcare NHS Trust, London, UK

**Keywords:** Objective, Assessment, Errors, Rectal cancer

## Abstract

**Background:**

Proficiency-based progression is key to analyzing and improving surgical performance. Objective assessment has demonstrated a direct link between operative performance and outcomes in laparoscopic surgery but not in robotics. There is current research to automate assessment processes with sensor data and machine learning. This requires granular, reliable annotations to train clinically implementable, trusted models, to improve patient safety.

**Aim:**

To evaluate objective skill and error tools in robotic rectal cancer surgery, to provide a granular validated dataset from which to train and test deep learning models.

**Methodology:**

A national, ethically approved, multicentre study, Video Analysis in Minimally Invasive Surgery (VAMIS) (ClinicalTrials.gov NCT05279287), recorded robotic-assisted total mesorectal excision (RTME). Recruited participants were pseudonymised and clinical data were collected. Operations were recorded and uploaded to Touch Surgery™ using the DS1 computer (Digital Technologies, a Medtronic company) and annotated by independent, blinded raters. Objective assessment employed error, Objective Clinical Human Reliability Analysis (OCHRA), Modifiable-Global Evaluative Assessment in Robotic Skills (M-GEARS) and TME performance tools. Correlational and multivariable regression analyses were performed, investigating associations between intraoperative skill and errors with clinical outcomes.

**Results:**

30 RTME operations were recorded, annotating 538 errors (median 13/operation). Major consequential errors were significantly associated with complications (*p* = 0.031). Weighted error variables, accounting for error severity, were significantly associated with increased odds of prolonged operative time (*p* = 0.025). Inter-rater reliability demonstrated an excellent matched error agreement percentage of two raters (mean agreement 90% (range 68–100%), after calibration sessions). OCHRA was significantly correlated with M-GEARS (*r* = − 0.54 to − 0.77, *p* < 0.001–0.002) and the RTME performance tool (*r* = 0.74, *p* = 0.007).

**Conclusion:**

This feasibility study validated the concept that granular error and skill annotations can be objectively measured and associated with clinical outcomes in robotic rectal cancer surgery. This is an important step for larger studies and in aiding the development of deep learning models to predict errors and skill.

**Supplementary Information:**

The online version contains supplementary material available at 10.1007/s00464-025-12393-x.

Colorectal cancer is the third commonest form of cancer, making up approximately 10% of all cancer diagnoses, and the second leading cause of cancer death worldwide [1]. The mainstay of treatment for these patients remains surgical excision. Professor Heald’s description of the “Holy Plane” [2] to follow embryological planes and totally excise the rectum with its mesentery, lymph nodes and appropriate vessels (total mesenteric excision (TME)), led to reduced local recurrence [3] and improved survival [4], highlighting the importance of high-quality surgical resections. However, much variation still exists within surgical resection quality [5, 6]. The need, therefore, for intraoperative surgical skill and errors to be measured objectively is apparent, with multiple studies associating surgical performance with patient outcomes [7, 8], including the quality of resection and histopathology in laparoscopic total mesenteric excision [6].

Analysing surgical performance through objective assessment is central to formative and summative processes involved in the concept of proficiency-based progression. However, many assessment tools are under-evaluated in minimal access surgery, and there is a distinct lack of scope within and between specialties [9]. Moreover, despite clinical researchers knowing the above, it is difficult to implement these tools within clinical practice and credentialing processes, and they are absent within most curricula. There is a need, therefore, to continue comprehensive development and evaluation of objective tools in key, high-stakes, operations.

The surgical research community has recognised these short comings and efforts continue, with much promise and drive towards automating these processes with robotic sensor data and machine learning models [10–13]. Artificial intelligence requires granular and reliable annotations to train trusted models before their implementation into clinical practice. If this is achieved, it would transform surgical training and intraoperative performance, providing real-time clinical feedback with intraoperative navigability of correct tissue planes and recognition or even anticipation of errors. In addition, it could automatically assess an operation for feedback and examination purposes, based on validated processes. Ultimately, these efforts aim to improve patient safety and outcomes.

The Video Analysis in Minimally Invasive Surgery (VAMIS) was an ethically approved clinical study, designed to capture video recorded minimal access surgery. The study aimed to evaluate objective assessment tools and their association with patient outcomes. This would provide a clinically validated database, with granular skill and error annotations in robotic-assisted total mesorectal excision (RTME), informing the future training and testing of computer vision and deep learning algorithms.

## Methodology

VAMIS is a national multicentre study (protocol on ClinicalTrials.gov NCT05279287) which gained Health Research Authority and Health and Care Research Wales (HCRW) approval with local Research Ethics Committee (REC) approval for national implementation (REC reference 22/EM/0046, IRAS project ID: 209024). It achieved NHS portfolio adoption; therefore, gaining support from local clinical research nurses. Four colorectal sites underwent local site set up with the installation of the Touch Surgery™ ecosystem, including its DS1 Computer from Digital Technologies, a Medtronic Company. This allowed recording, through the RedactOR™, which operates locally in real time to identify ‘camera-out’ events and applies pixelation to safeguard privacy, thereby, facilitating blinded, independent rating on the Touch Surgery™ annotation platform. Patient participants, over the age of 18, who were undergoing an elective robotic anterior resection for rectal cancer were recruited and assigned a pseudonymised identity (ID) by the local research team.

### Skill and error annotation tools

Blinded, independent, single rater, retrospective video analysis was implemented, utilising three broad skill methodologies: generic skill rating, using the validated Modifiable-GEARS tool [14], a procedure-specific task analysis sheet (Supplementary Table 1) with a competency assessment TME performance tool [6] (Supplementary Fig. 1). Error annotations implemented the validated objective clinical human reliability analysis (OCHRA) [6, 15] alongside the European Association of Endoscopic Surgery (EAES) Likert scale error severity classification system [16] (Supplementary Table 2). An additional classification system was created: “Pre-error”, representing a near miss or non-consequential error (EAES severity 1) and “Error” (EAES severity 2 to 5), representing any error, from minor to major consequential errors. This was to simplify classification in efforts to aid deep learning model development through binary outcomes as opposed to ordinal.

### Data analysis

#### Inter-rater reliability

Inter-rater reliability was assessed by comparing surgical videos between two trained raters, one a senior surgical resident and the other a credentialed colorectal consultant. Both have experience with OCHRA and were trained to use it by the laparoscopic TME dataset primary author (NC) and corresponding author (NKF) who has developed and implemented OCHRA over the past two decades. As ranking statistical analyses only correlate the number of errors between raters, they miss whether the raters are annotating the same errors; therefore, a “matched error agreement percentage” was performed before and after calibration sessions. These sessions were designed to set thresholds and align annotations between raters, with the percentage agreement calculated as below:

$${\text{Matched error agreement percentage}} = \frac{{{\text{Both raters have annotated }}\& {\text{ agree on the error}}}}{{{\text{Both raters have annotated }}\& {\text{ agree on the error }} + {\text{ Missed error or disagreement}}}} \times 100$$

#### Concurrent validity

Concurrent validity, or Messick’s validity domain of “Relationship to other variables”, between skill and error objective assessment tools was analysed with Pearson’s test.

#### Clinical validity

Clinical endpoints were captured including 30-day morbidity, graded with the Clavien-Dindo classification, reoperation, anastomotic leak and resection margins.

Multivariable regression analyses, accounting for confounders, were used to investigate the association, predictive validity, of annotated skills and errors with patient outcomes (Supplementary Tables 3 & 4). Different error variables were created to provide in-depth analyses, rather than simply using the total number. The rationale for this was perhaps an operation may be longer due to patient and tumour factors; therefore, the total number of errors is greater. Importantly, not all errors have the same consequence severity, and therefore, in theory, should have a varying impact on patient outcomes. In anticipation that there could be videos with a lower frequency of errors, but more severe, the error counts were transformed to reflect this into linear and exponentially weighted scores. Instrument clashes and needles that were held out of view were annotated to investigate the association with skill and outcomes.

Three error groups were created:

(1) Total errors—with no suffix as below.

(2) Errors excluding instrument clashes and needles out of view (“no_clash_no_needle”).

(3) Only instrument clashes and needles out of view (“clash_needle_only”).

The “no_clash_no_needle group” was hypothesised to be the most representative of “important” errors captured, given that clashes and needles out of view were unlikely to be consequential, and they were numerous. When referring to errors in the Results and Discussion, this will be the default group unless stipulated otherwise.

## Results

Thirty patient participants with rectal cancer were recruited in this study including 20 males (66.67%), with a mean age of 69 years (range 53–81), mean BMI of 26.6 (range 17.5–40.4). Twenty-two (73.3%) participants had an ASA II and the remaining 8 (26.7%) ASA III. Six (20%) participants were recorded as having no other co-morbidities according to the Charlson co-morbidity scoring index. Twenty-four (80%) participants were recorded as suffering from significant co-morbidities. Twenty-three (76.7%) participants were White British, 2 (6.7%) White Irish, 2 Asian Indian or Asian British, two preferred not to say, one (3.3%) Any other Asian background. Seventeen (56.7%) participants had a high (partial) TME resection, 10 (33.3%) low anterior resection and 3 (10%) had an ultra-low anterior resection. Nine participants (30%) had a stoma formed, 21 (70%) did not. Twenty-six (86.7%) had no neoadjuvant treatment, 4 (13.3%) had chemotherapy. There were 7 senior surgeon participants with a median robotic case load of 300 (mean 380, range 10–800), with a median laparoscopic case load of 867 (mean 439, range 200–1500) (Supplementary Table 5). Three trainees were recorded as operating supervised. The median length of stay was 5 days (range 2–30 days). Ten (33.3%) participants suffered a complication within 30 days of the operation. Seven (23.3%) participants suffered a complication on the same admission, 6 (20%) Clavien-Dindo II severity and 1 (3.3%) Clavien-Dindo IIIb. Three (10%) participants were readmitted within thirty days, one (3.3%) with a Clavien-Dindo IIIa severity complication, and two (6.7%) with a Clavien-Dindo IIIb complication. Twenty-eight (93.3%) had a complete (R0) resection, with 2 (6.7%) having an incomplete (R1) resection.

Three (10%) anastomotic leaks were reported, one (3.3%) required endoscopic treatment and two (6.7%) had operative interventions. Two (6.7%) patients suffered an ileus, one (3.3%) had a high stoma output and four of the ten complications had no descriptions specified.

A total of 6,635 min, 110.5 h, 4.61 days of operative video was analysed, taking 10, 867 min, 181.12 h, 7.55 days to annotate. The median intra-corporeal operative time was 216 min (range 104–438). The median time taken to annotate each video was 345 min (range 130–812).

The overall total number of errors (Supplementary Table 6) was 1540 (median 43, range 9–191), with a median error per minute of 0.2, or every 5 min. The total number of errors in the group, excluding instrument clashes and needles out of view (“no_clash_no_needle” group), was 538 (median 13, range 3–91), or one error every 15.6 min. There were 279 near misses (median 6, range 0–56) (EAES 1 no clash no needle), 256 (median 7, range 0–35) minor consequential (EAES 2 no clash no needle), and 3 (mean 0.1, range 0–1) major consequential (EAES 3 no clash no needle) errors.

Observing the clashes and needles out of view only (“clash_needle_only”) group, there were 1002 (median 26, range 5–100), errors. There were instrument clashes or needles out of view 0.14 times per minute or every 7 min.

**Fig. 1:**
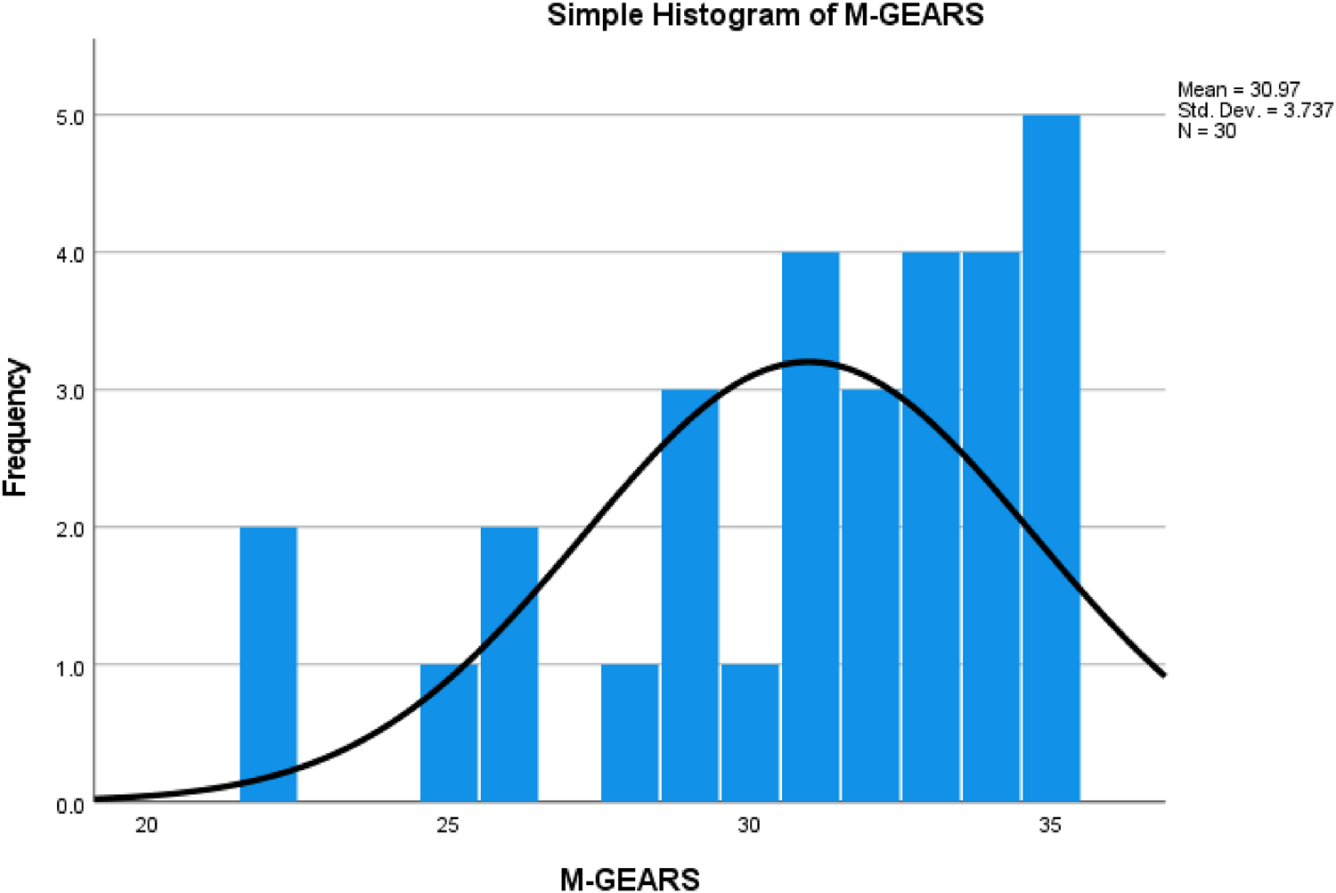
Histogram of modifiable-GEARS score frequencies

Modifiable-GEARS median scores were 32/35 (91.43%, range 22–35) (Fig. 1). The TME performance tool median percentage score was 82.81 (range 60.4–100%).

Observing the new binary severity classification score group (*n* = 24) of “Pre-Error” and “Error”. There was a total of 919 (median 32.5, range 7–109) pre-errors and 196 (median 6.5 range 0–18) consequential errors.

The following results look at the coded errors for each OCHRA domain. The top three most frequent technical errors for (Table 1) were “Inappropriate grasping/blunt handling of structure” (82/545 (15%)), “Diathermy/dissection in wrong tissue plane” (78/545 (14.3%)) and “Instrument clash due to poor control of instrument” with consequences (54/545 (9.9%)).

**Table 1:** Technical errors by frequency

Technical errors		
	Frequency	Percent
Inappropriate grasping/blunt handling of other structure	82	15.0
Diathermy/dissection in wrong tissue plane	78	14.3
Instrument clash due to poor control of instrument	54	9.9
Inappropriate use of diathermy/cutting (tip of instrument visualised)	52	9.5
Poor visualisation of instrument tip	40	7.3
Traction applied with too much tension	35	6.4
Instrument out of view	32	5.9
Too much blunt force applied to tissue	31	5.7
Dissection performed in wrong direction	18	3.3
Other	17	3.1
Poor instrument control	16	2.9
Poor camera view	13	2.4
Use of inappropriate instrument to retract	11	2.0
Inadequate pedicalisation	10	1.8
Instrument clash due to inadequate view	10	1.8
Overshoot of instrument movement	10	1.8
Inappropriately placed clips	9	1.7
Operating with poor vision	9	1.7
Too much/little energy applied with instrument	6	1.1
Instrument clash due to narrow space	4	0.7
Traction applied with too little tension	4	0.7
Instrument clash due to difficult view, e.g. port placement	1	0.2
Needle driven with force/inappropriately	1	0.2
Needle not sheathed or retrieved safely	1	0.2
Use of inappropriate instrument to dissect	1	0.2
Total	545	100.0

The error event (consequence) domain (Table 2), the top two consequences were “Bleeding (ooze)” (20/545 (36.9%)) and “Risk of injury to other structure” (165/545 (30.3%)). The next commonest, “Delay in progress of procedure of procedure” and “Risk of mesorectal injury”, were far less frequent.

**Table 2:** Event error frequencies

Event error (consequence) frequencies		
	Frequency	Percent
Bleeding (ooze)	201	36.9
Risk of injury to other structure	165	30.3
Delay in progress of procedure	34	6.2
Risk of mesorectal injury	29	5.3
Injury to other structure	18	3.3
Injury to pelvic nerves	15	2.8
Bleeding (significant/pulsatile)	14	2.6
Mesorectal injury into mesorectal fat	14	2.6
Risk of injury to pelvic nerve	13	2.4
Avulsion of tissue	11	2.0
None	8	1.5
Other	8	1.5
Object dropped	6	1.1
Mesorectal injury breach of fascia only	2	0.4
Misaligned anastomosis	2	0.4
Blunt bowel injury	1	0.2
Diathermy burn to viscus	1	0.2
Injury to parietal pelvic fascia	1	0.2
Mesorectal injury into rectal musculature	1	0.2
Oncological compromise of operation	1	0.2

In the external error mode domain (Table 3), the two commonest were “Step is done in wrong orientation/direction/point in space” (238/545 (43.7%)) and “Step is done with too much force/speed/depth/distance/time/rotation” (199/545 (36.5%)).

**Table 3:** External error mode frequencies

External error mode frequencies		
	Frequency	Percent
Step is done in wrong orientation/direction/point in space	238	43.7
Step is done with too much force/speed/depth/distance/time/rotation	199	36.5
Step is done on/with the wrong object	46	8.4
Step is done with too little force/speed/depth/distance/time/rotation	17	3.1
Step is partially completed	17	3.1
Step is not done	11	2.0
Step is done out of sequence	10	1.8
Step is repeated	5	0.9
Unknown	1	0.2

The most frequently used instruments enacting the errors were “Monopolar shears” (264/545 (48.4%)) and “Grasper—blunt” (164/545 (30.1%)) (Supplementary Table 7). The most frequent recovery mechanisms (Supplementary Table 8) from an error were “Corrective action within subtask” (298/545 (54.7%)) and “Continue uninterrupted” (210/545 (32.3%)).

### Inter-rater reliability

11 videos were analysed by two independent, blinded, trained raters. Matched error agreement percentages are provided in Table 4. Pre-calibration inter-rater reliability median agreement was 35% (range 17–77%). Post-calibration sessions the median agreement was 90% (range 68–100%).

**Table 4:** Matched error percentage agreement inter-rater reliability analysis

Video	Pre-calibration	Post-calibration	Post 2nd calibration with chief investigator
1	0.77	1.00	N/A
2	0.29	1.00	N/A
3	0.19	0.90	N/A
4	0.52	0.80	0.92
5	0.47	0.68	1.00
6	0.40	0.90	1.00
7	0.17	0.72	1.00
8	0.42	1.00	N/A
9	0.15	0.92	1.00
10	0.19	0.88	1.00
11	0.35	1.00	N/A

### Concurrent validity between skill and error objective assessment tools

Significant moderate to strong inverse correlation (*r* = − 0.54 to − 0.77, *p* < 0.001 to 0.002) (Supplementary Table 9) was demonstrated between multiple error variables and M-GEARS. A significant strong correlation (*r* = 0.74, *p* = 0.007) was demonstrated between Modifiable-GEARS and the procedure-specific TME performance tool.

There was a significant correlation with EAES 1 and EAES 2 (*r* = 0.82, *p* < 0.001). A strong correlation was demonstrated between “Pre-error” and “Error” binary severity classification (*r* = 0.71, *p* < 0.001).

Table 5 demonstrates that there was one near miss enacted for every minor consequential error, 93 near misses for every major consequential error and 4.69 “Pre-errors” per “Error” of any consequence.

**Table 5:** Ratio of error severity variables RTME dataset

Test	Variable	Variables	Ratio	Interpretation
Division	EAES 1	EAES 2	1.09	No significant difference comparing means with independent samples t test *p* = 0.154. Demonstrating that for every near miss there is a minor consequential error
Division	EAES 1	EAES 3	93	93 near misses to every 1 major consequential error
Division	EAES 2	EAES 3	85.33	85 minor consequential to every 1 major consequential error
Division	Pre-error	Error	4.69	For every 4.69 pre-errors there is a consequential error of any severity

### Clinical validation of annotated error and skill scores with patient outcome data

Regression models (Supplementary Tables 10 & 11) demonstrated that multiple OCHRA error variables were significantly associated, or approached significance, with increased odds of peri- and post-operative metrics and patient outcomes. EAES 3 (major consequential) severity errors were significantly associated with same admission and readmission complications (*p* = 0.031). Multiple error variables approached significance with same admission Clavien-Dindo complications (*p* = 0.061–0.096) and EAES 3 severity errors approached significance (*p* = 0.055) with length of stay.

### Instrument clashes and needles out of view associations with skill and outcomes

A moderate significant inverse correlation was demonstrated between M-GEARS and clashes and needles out of view (*r* = − 0.65, *p* < 0.001). A strong significant correlation was associated between “Error” severity classification and clashes and needles out of view (*r* = 0.78, *p* < 0.001) (Supplementary Table 9). Clashes and needles out of view were significantly inversely associated with operative time (*p* = 0.025) and approached significance with “same admission complications (*p* = 0.083) (Supplementary Table 11).

## Discussion

The robotic TME study arm of the Video Analysis in Minimally Invasive Surgery (VAMIS) study has demonstrated significant associations between observed intraoperative errors and peri- and post-operative patient outcomes. In addition, concurrent validity with strong inter-rater reliability for skill and error tools respectively has been established. This robotic dataset complements the originally evaluated laparoscopic TME study and builds a validated database from which to train and test deep learning skill and error prediction models.

There was no significant difference between the number of EAES 1 near misses and EAES 2 minor consequential errors. Observing the binary severity classification, most errors are minor, with 82.4% being pre-errors. Of note, the sum total is less in the binary severity classification due to a later implementation of the classification system within the analysis. Implemented objective assessment tools suggested an overall high skill level with M-GEARS median scores of 32/35 (91.4%) and a positive skew (Fig. 1), and the TME performance tool median score of 82.8%.

The two commonest technical errors were “Inappropriate grasping/blunt handling of other structure” and “Diathermy/dissection within the wrong tissue plane”, which reflect the commonest instruments enacting errors, i.e. “Grasper—blunt” and “Monopolar Shears”. The most frequent consequences were all minor: “Bleeding (ooze)”, “Risk of injury to other structure”, and “Delay in progress of procedure” which correlate with the number of EAES 1 and 2 annotations. Whilst reviewing “how” the error is enacted, the two commonest were “Step is done in wrong orientation/direction/point in space” (43.7%) and “Step is done with too much force/speed/depth/distance/time/rotation” (36.5%). Interestingly, this reflects the top consequences and external error modes observed within other datasets and likely represents common themes within minimal access surgery. It is not only a key example of how OCHRA can highlight domains for formative feedback but also inform us of possible high-yield areas to focus AI development.

We chose to implement three manual objective assessment tools to fully capture the skill of the surgeon. Global rating scales capture how well the surgeon utilises instruments and the fluency or nuances that can be difficult to describe within expert versus novice gestures, but are inherently obvious to the observer. This is missed by the other tools. Procedure-specific assesses if the key steps are performed and the correct execution of them, whilst both these tools will miss granular error analysis. These tools were chosen as M-GEARS is the most validated robotic global rating scale tool [9], the procedure-specific and OCHRA tools were comprehensively evaluated with strong predictive validity in laparoscopic rectal cancer, and by our group, so deemed appropriate to utilise in robotic TME.

Strong intra- and inter-rater and reliability has previously been demonstrated for OCHRA [6, 15, 17, 18] however, this correlated with the total error counts or ranks. This works well for scoring systems in objective assessment tools like M-GEARS and the TME performance tool, but it is more important that the same errors are being rated. A strong correlation between the total number of errors would only indicate an agreement on the skill level of the surgeon. Although useful, the real hope for OCHRA is its use in training AI models to predict and recognise errors. For models to be clinically implemented they must be trusted and developed from comprehensively evaluated error methodology which has ensured excellent inter-rater reliability of the errors that they annotate. Therefore, a decision was made to use a matched error agreement percentage for inter-rater reliability [19, 20]. This created the idea of calibration sessions, which initially demonstrated low reliability, but increased to a strong matched agreement after these sessions. This was often due to errors being missed, rather than disagreement, which is hardly surprising given that OCHRA annotation of one video took a median time of 3 hours and 45 minutes to complete. This is important to note; as we move forward evaluating large datasets, it suggests that each video should be annotated by more than one rater, as there will inevitably be missed errors, and therefore, skew what AI models learn. It may also incorrectly reduce models’ prediction accuracies. Imagine an evaluated model that is then used on an unseen surgery, using ground truth annotations from only one rater. The AI model may identify errors that have not been annotated, and despite correctly predicting it, the accuracy will be incorrectly lowered. This could be likened to a type II statistical error. It is imperative that efforts are made to fully validate datasets, including this one, with multiple raters and calibration sessions, despite their time-consuming nature. It also encourages us to think of alternative strategies, such as annotating clipped videos of key phases.

The most difficult challenge in these sessions was setting the threshold of certain errors. One such scenario was when a surgeon retracts the tissue to the ideal tension to dissect effectively; however, it causes minor bleeding necessitating corrective action in the process. The surgeon’s skill here is high and they have been operating appropriately, but one rater would annotate the error as a minor consequential error, and the other may not. Calibration sessions are key in identifying these areas and aligning ratings. Training AI how to distinguish this is another matter altogether. Again, this requires multiple expert surgeon annotators, representing a huge challenge to achieve this internationally, across thousands of videos, and multiple specialty operations. The concept of non-surgical crowd-sourced raters has been implemented for other skill assessments to enable rapid, multi-rater analysis. However, the granularity and understanding required to implement OCHRA methodology, including the nuances of surgical gestures and tasks, likely excludes the use of non-surgical crowd-sourced raters.

The regression analyses demonstrated the predictive validity of major consequential errors being significantly associated with complications and approached significance with length of stay. Other error variables also approached significance with Clavien–Dindo complications. The sample size here is small at only 30 operations; with further analyses this may demonstrate that there is a significant association between many of these variables, as with Curtis et al. in their 176 analysed cases. With this small dataset it is more likely to fail to reject the null hypothesis, that there is no association between skill and errors with patient outcomes. Interestingly, instrument clashes and needles out of view demonstrated an inverse association with operative time, i.e. faster surgery resulted in more clashes, perhaps indicating that a surgeon is rushing. Time, as surgeons know, is a crude measurement of surgical performance.

Despite the promising results, regression analyses can miss the nuance captured by direct observation and OCHRA annotation. In one operation the rectum was not fully mobilised, necessitating a redo stapled excision, resulting in two specimens and technically an R1 histology. In the regression analyses, there was no association with resection margins and errors; however, this is a key learning moment that can be used in formative training sessions. We advocate that key video clips are highlighted within annotating processes, as has been done with this dataset, to be used for future teaching purposes.

M-GEARS and errors were significant and strongly correlated as were near misses and minor consequential errors, indicating that more consequential errors occur in surgeries with more near misses.

As with the laparoscopic TME data [6], the VAMIS RTME study [6] again demonstrates an unfavourable ratio, when comparing surgery to other high-risk industries, of 93 near misses for every major consequential error [6].

### Challenges, limitations and future directions

This was a single-blinded, multi-institutional, observational, non-randomised, prospective study. A median time of 353 days (range 55–747) between first approach to site and study activation, and a further median of 115 days (range 28–116) from study activation to the first recording. When adding the five months of ethical approval time it took over 2 years from initiation until the first video analysis, and therefore serves as the main reason for the relatively small sample size. This highlights the challenges of implementing digital technology within the clinical healthcare system, and it is a topic that deserves attention with the inevitable explosion of novel technologies.

The sample population was heterogeneous with high and low rectal cancers. However, as a feasibility study it was still deemed appropriate to analyse together, along with accounting for this as a confounder in the regression analyses.

Following VAMIS, additional research will require larger, multi-institutional datasets with standardised patient outcome metrics. Preferably, this would be in the form of randomised control trials comparing an intervention group of surgeons who have defined formative assessments by raters who have undergone robotic-specific Training-The-Trainer courses, such as has been done in the Colorobotica curriculum [21]. Proficiency-based progression CUSUM analyses could be performed as was done in LapCo [22, 23] to indicate the level at which the trainee gains competency. At this point, blinded, independent experts could use the validated summative objective assessment tools to accredit surgeons through unedited video analysis. This would also help formally benchmark these tools as part of curricula. Moreover, it would provide a large, expertly validated dataset for artificial intelligence models to be developed with a higher level of trust in the ground truth annotation, and so more likely to be implementable into the clinical setting.

Manual annotation carries its own challenges including the need for further expert consensus on OCHRA and error severity modifications, threshold setting for multiple specialty operations as well as multiple raters on large datasets, which currently is demonstrably not feasible outside of the research setting. This is a huge challenge, and the surgical community is committed to finding a way to do this swiftly, with expert consensuses providing frameworks to achieve these goals [24, 25].

This dataset is small but can contribute to the initial development and evaluation of AI models to lay a foundation for future research. Tackling the issue of large datasets requires collaboration between industry, computer scientists, and clinicians within international healthcare systems to address issues with consent, ethics and data sharing. As a pragmatic, feasibility study, Table 6 has several recommendations to aid further research.

**Table 6:** Challenges and recommendations from VAMIS

Challenges highlighted in VAMIS	Example	Recommendation
Need for larger datasets	VAMIS is a small, unpowered data set with some results approaching significance. A type II error is possible	Further large collaborative research efforts to be sought, preferably within a national training programme such as LapCo to validate objective assessment tools and the analysed datasets
OCHRA inter-rater reliability	OCHRA is time consuming, and IRR analyses is yet to be achieved within VAMIS RARP and was only performed on 11 videos in VAMIS RTME OCHRA requires multiple raters as there are often missed errors that still have rater agreement	Consensus with statisticians and expert surgeons on the best way to perform IRR for OCHRA, with scoring rankings as well as matched percentage error agreement Efforts such as SAGES Critical View of Safety challenge demonstrate how multiple, international, trained surgeon annotators can label multiple clips. Similar efforts could be made within error annotation and be achieved on a virtual platform
Implementing research and technology within healthcare systems	Implementing ethics and digital technology encountered significant delays. Ethical approval protocols are unlikely to change, and positively, it is nationally approved process. However, integrating digital technology had huge hurdles with every NHS site having different implementation processes. One site still has not completed this process 3 years on. This process, anecdotally, disincentivised many local researchers and affected subsequent surgeon and patient participant recruitment	Consensus from key NHS research stakeholders is required to identify and create strategies on the safest and most efficient way to integrate digital technology whilst evaluating their safety This research group aims to highlight this in a joint report article highlighting the issue, potentially between VAMIS researchers and industry, e.g. Digital Technologies, a Medtronic Company
OCHRA definitions	There is a need for modifications in each domain	Further multi-specialty expert consensus to agree on the potential creation of truly generic OCHRA domains to allow coding in multiple operations without the need for (significant) modification
EAES error classification	There is possible subjectivity within the classification systems, reducing inter-rater reliability	The EAES error classification working group could modify the error classifications including to intraoperative sequelae, rather than post-operative
Error variable data analyses	This research has highlighted the weighting, linear and exponential, of errors. The regression models suggest that this is key to understanding errors, with EAES 3 being linked to complications when EAES 1 and 2 weren’t. This seems obvious but has been demonstrated for the first time	Multi-specialty expert consensus on how best to move forward with error variable analyses Larger dataset analyses are required to fully understand the impact of errors on patient outcomes
Development and evaluation of deep learning models to predict skill and errors	There is a dearth of scope and evaluation of artificial intelligence assessment tools which are in their infancy	This dataset should be utilised and discussed with computer scientists, to help the development and evaluation of deep learning models to predict skill and errors, building on our research on basic robotic suturing in RARP^584^

## Conclusion

The Video Analysis in Minimally Invasive Surgery (VAMIS) robotic TME study arm has demonstrated promising results in the association of observed intraoperative skill and errors, using objective assessment tools, with peri- and post-operative patient outcome metrics. Further research is required to establish error thresholds, reliability analyses and the development of automated assessment deep learning algorithms to predict skill and errors. The results act as a baseline from which to gain expert consensus on ground truth ratings to further evaluate annotation processes and automated skill assessment in robotic surgery.

## Supplementary Information

Below is the link to the electronic supplementary material.

Supplementary file1 (DOCX 548 KB)
