# Supplementary material for: Association of skill and errors with outcomes in robotic rectal cancer surgery
Source: Surg Endosc. 2025 Dec 10;40(3):2085–94. doi: 10.1007/s00464-025-12393-x (PMC12971788; doi:10.1007/s00464-025-12393-x)
Supplement: Supplementary file 1 — Supplementary file1 (DOCX 548 KB) [file 464_2025_12393_MOESM1_ESM.docx]

**Supplementary materials**

Table of contents

[Figure 1 Laparoscopic Total Mesenteric Excision performance tool (LapTMEpt) 2](#_Toc201421134)

[Figure 2 EAES error classification 2](#_Toc201421135)

[Table 1 TME Task analysis sheet 2](#_Toc201421123)

[Table 2 OCHRA RTME error methodology domains 3](#_Toc201421124)

[Table 3 Outcome (dependent) variables and models analysed 7](#_Toc201421125)

[Table 4 Generic predictor variables for regression models 8](#_Toc201421126)

[Table 5 Number of videos analysed per surgeon and their caseload 10](#_Toc201421127)

[Table 6 Descriptive statistics of error group frequencies 10](#_Toc201421128)

[Table 7 Instrument enacting error frequencies 12](#_Toc201421129)

[Table 8 Recovery mechanism frequencies 12](#_Toc201421130)

[Table 9 Correlation between skill and error scores RTME dataset 13](#_Toc201421131)

[Table 10 VAMIS RTME Summary of significant, and approaching significance, regression models 14](#_Toc201421132)

[Table 11 VAMIS RTME Regression models with statistically significant results when adjusting for confounders 17](#_Toc201421133)

Supplementary Figures:


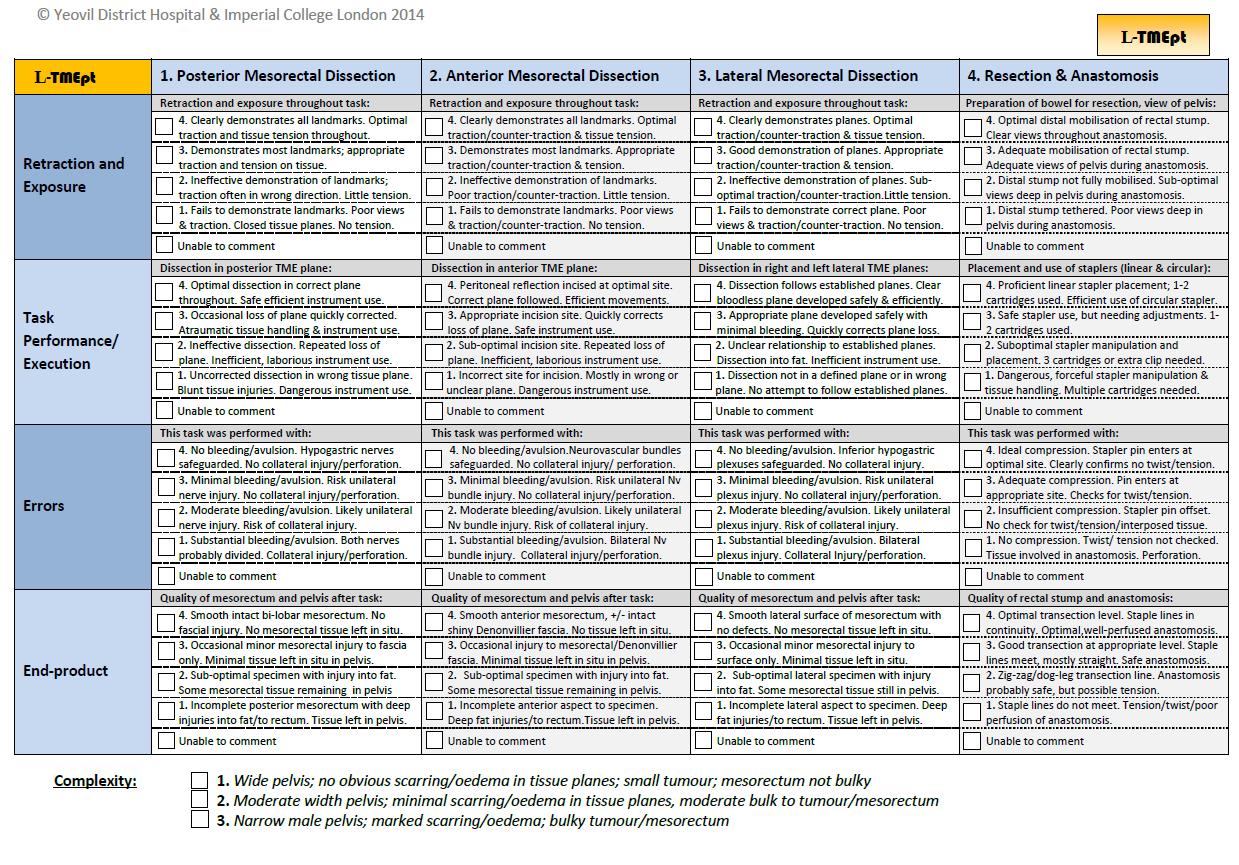


Figure 1 Laparoscopic Total Mesenteric Excision performance tool (LapTMEpt)


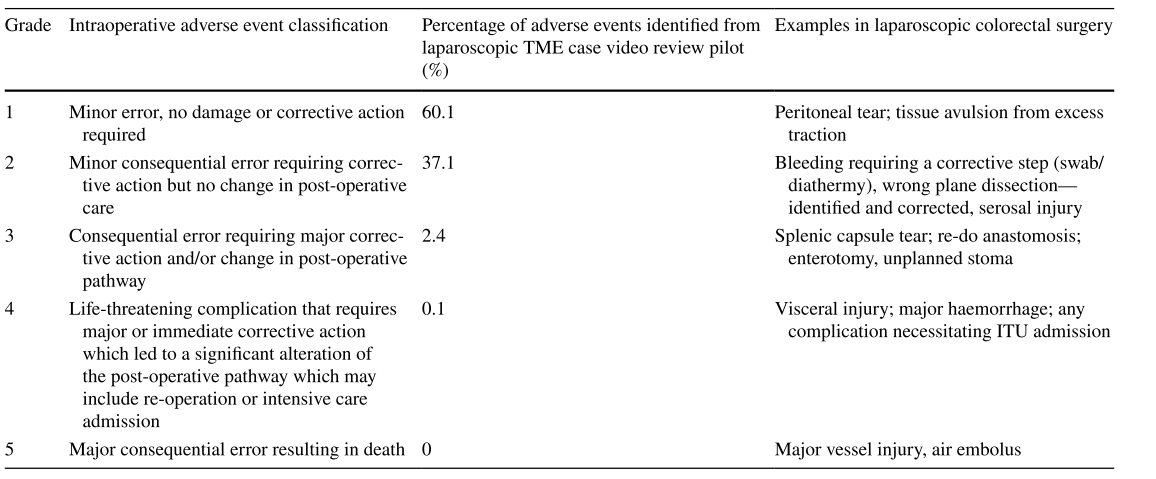


Figure 2 EAES error classification

Supplementary tables:

Table 1 TME Task analysis sheet

| **Phase (Task)** |
| --- |
| 1. Preparation of patient |
| 2. Exposure of operating field |
| 3. Division of vascular pedicles |
| 4. Mobilisation of descending colon |
| 5. Mobilisation of splenic flexure |
| 6. Posterior mesorectal dissection |
| 7. Anterior mesorectal dissection |
| 8. Completion TME |
| 9. Division and anastomosis |
| 10. Completion |
| **Phase metrics (subtask)** |
| 2a. Port insertion pneumoperitoneum |
| 2b. Preparation of operating field |
| 2c. Division of lateral attachments to sigmoid colon |
| 3a. Dissection around IMA incising peritoneum over sacral promontory |
| 3b. Division of IMA |
| 3c. Dissection around IMV- division of peritoneum over IMV |
| 3d. Division of IMV |
| 4a. Medial to lateral mobilisation of colon |
| 4b. Lateral Mobilisation of colon |
| 5a. Medial mobilisation of splenic flexure |
| 5b. Lateral mobilisation of splenic flexure |
| 5c. Inferior mobilisation of splenic flexure |
| 6a. Posterior mesorectal dissection |
| 6b. Right postero-lateral mesorectal dissection |
| 6c. Left postero-lateral mesorectal dissection |
| 7a. Division of anterior peritoneal reflection |
| 7b. Anterior mesorectal dissection |
| 8a. Distal posterior TME |
| 8b. Distal anterior and lateral TME |
| 9a. Cross-stapling of rectum |
| 9b. Evacuation of specimen |
| 9c. Anastomosis |
| 10a. Drain placement |
| 10b. Stoma |
| 10c. Closure of wounds and laparoscopic port sites |

Table 2 OCHRA RTME error methodology domains

| **Technical Errors** |
| --- |
| Cutting without lifting tissues from underlying structures |
| Diathermy/dissection in wrong tissue plane |
| Dissection performed in wrong direction |
| Excess force on needle |
| Inadequate pedicalisation |
| Inappropriate grasping / blunt handling of other structure |
| Inappropriate handling of tumour |
| Inappropriate use of diathermy / cutting (tip of instrument visualised) |
| Inappropriately placed clips |
| Instrument applied with too little distance to structure |
| Instrument clash due to difficult view e.g. port placement |
| Instrument clash due to inadequate view |
| Instrument clash due to narrow space |
| Instrument clash due to poor control of instrument |
| Instrument out of view |
| Needle bite too deep |
| Needle bite too shallow |
| Needle driven with force/inappropriately |
| Needle drives too close or too far apart |
| Needle grasped at needle tip |
| Needle not sheathed or retrieved safely |
| Needle out of view |
| Operating with poor vision |
| Other |
| Overshoot of instrument movement |
| Poor camera control |
| Poor camera view |
| Poor instrument control |
| Poor visualisation of instrument tip |
| Suture entanglement/thread caught in instrument |
| Too much blunt force applied to tissue |
| Too much/little energy applied with instrument |
| Traction applied in wrong direction |
| Traction applied with too little tension |
| Traction applied with too much tension |
| Use of inappropriate instrument to retract |
| **Consequences** |
| Avulsion of tissue |
| Bleeding (ooze) |
| Bleeding (significant / pulsatile) |
| Blunt bowel injury |
| Blunting of needle |
| Breaking needle tip |
| Delay in progress of procedure |
| Diathermy burn to other structure |
| Diathermy burn to viscus |
| Injury to other structure |
| Injury to parietal pelvic fascia |
| Injury to pelvic nerves |
| Injury to ureter |
| Mesorectal injury breach of fascia only |
| Mesorectal injury exposing rectal adventitia |
| Mesorectal injury into mesorectal fat |
| Mesorectal injury into rectal musculature |
| Misaligned anastomosis |
| Misplacing the needle |
| None |
| Object dropped |
| Oncological compromise of operation |
| Other |
| Perforating bowel injury |
| Port dislocation |
| Rectal perforation |
| Risk of injury to other structure |
| Risk of injury to pelvic nerve |
| Risk of mesorectal injury |
| Sharp injury to other structure |
| Sharp injury to viscus |
| **External error mode** |
| Step is not done |
| Step is partially completed |
| Step is repeated |
| Step is done out of sequence |
| Step is done with too much force / speed / depth / distance / time / rotation |
| Step is done with too little force / speed / depth / distance / time / rotation |
| Step is done in wrong orientation/direction/point in space |
| Step is done on/with the wrong object |
| **Instrument** |
| Bowel clamp |
| Camera |
| Finger |
| Grasper - blunt |
| Grasper- fine |
| Hemoloc/metal clip |
| Hook |
| Laparoscopic blunt grasper |
| Laparoscopic fine grasper |
| Laparoscopic needle driver |
| Laparoscopic scissors |
| Monopolar shears |
| Needle holder |
| Retractor |
| Robotic stapler |
| Suction |
| Swab |
| Vessel sealer/synchroseal |
| **Recovery mechanism** |
| Continue uninterrupted |
| Perform step previously omitted |
| Requires repetition of step |
| Corrective action within subtask e.g. adjust hold/visualisation/dissection plane |
| Change in subtask/sequence |
| Additional action |
| Port adjustment/replacement |
| Camera adjustment/clean |
| **EAES Severity** |
| 1. Near miss/Minor non-consequential error, no damage or corrective action required e.g. peritoneal tear, avulsion from excess traction |
| 2. Minor consequential error requiring corrective action but no change to operative in post-op care e.g. bleeding requiring a corrective step (swab/diathermy), wrong tissue plane dissection identified and corrected, serosal injury |
| 3. Consequential error requiring major corrective action and/or change to post-op pathway e.g. splenic capsule tear, redo anastomotic enterotomy, unplanned stoma |
| 4. Life-threatening complication that requires major or immediate corrective action which led to significant alteration of the post-op pathway which may include reoperation or ITU e.g. visceral injury, major haemorrhage, any complication necessitating ITU |
| 5. Major error resulting in death e.g. air embolus, major vessel injury |
| **Pre-error/error** |
| 0: Pre-error |
| 1: Error |

Table 3 Outcome (dependent) variables and models analysed

| **Role** | **Variable** | **Variable Type** | **Model** |
| --- | --- | --- | --- |
| Outcome | LOS | Numeric | Linear |
| Outcome | Time_of_operative_video_(minutes)_minus_the_extracorporeal_phase | Numeric | Linear |
| Outcome | Same_admission_HIGHEST_Clavien_Dindo_classification_ordinal | Ordinal | Ordinal |
| Outcome | Readmission_highest_Clavien_Dindo_classification_ordinal | Ordinal | Ordinal |
| Outcome | Same_admission_complication_binary | Binary | Logistic |
| Outcome | 30_day_readmission_binary | Binary | Logistic |
| Outcome | Resection_margin | Binary | Logistic |
| **Role** | **Additional variables trialled** | **Type** | **Model** |
| Outcome | Any_Clavien_Dindo_complication_ordinal | Ordinal | Ordinal |

Table 4 Generic predictor variables for regression models

| **Generic Predictor variables** | **With or without confounders** |
| --- | --- |
| Total_Error_number | none |
| Error/minute | none |
| Linear_weighted_sum_errors | none |
| Linear_weighted_sum_errorsminute | none |
| Exp_weighted_sum_errors | none |
| Exp_weighted_sum_errorsminute | none |
| Total_Error_no_clash_no_needle | none |
| Errorsminute_no_clash_no_needle | none |
| Linear_weighted_sum_errors_no_clash_no_needle | none |
| Linear_weighted_sum_errorsminute_no_clash_no_needle | none |
| Exp_weighted_sum_errors_no_clash_no_needle | none |
| Exp_weighted_sum_errorsminute_no_clash_no_needle | none |
| Total_Error_number_clash_needle_only | none |
| Errorsminute_clash_needle_only | none |
| Linear_weighted_sum_errors_clash_needle_only | none |
| Linear_weighted_sum_errorsminute_clash_needle_only | none |
| MGEARS | none |
| MGEARS_percentage | none |
| Total_Error_number | BMI, age, comorbidity, Neoadjuvant Rx, operation type (High or Low anterior resection) |
| Error/minute | BMI, age, comorbidity, Neoadjuvant Rx, operation type (High or Low anterior resection) |
| Linear_weighted_sum_errors | BMI, age, comorbidity, Neoadjuvant Rx, operation type (High or Low anterior resection) |
| Linear_weighted_sum_errorsminute | BMI, age, comorbidity, Neoadjuvant Rx, operation type (High or Low anterior resection) |
| Exp_weighted_sum_errors | BMI, age, comorbidity, Neoadjuvant Rx, operation type (High or Low anterior resection) |
| Exp_weighted_sum_errorsminute | BMI, age, comorbidity, Neoadjuvant Rx, operation type (High or Low anterior resection) |
| Total_Error_no_clash_no_needle | BMI, age, comorbidity, Neoadjuvant Rx, operation type (High or Low anterior resection) |
| Errorsminute_no_clash_no_needle | BMI, age, comorbidity, Neoadjuvant Rx, operation type (High or Low anterior resection) |
| Linear_weighted_sum_errors_no_clash_no_needle | BMI, age, comorbidity, Neoadjuvant Rx, operation type (High or Low anterior resection) |
| Linear_weighted_sum_errorsminute_no_clash_no_needle | BMI, age, comorbidity, Neoadjuvant Rx, operation type (High or Low anterior resection) |
| Exp_weighted_sum_errors_no_clash_no_needle | BMI, age, comorbidity, Neoadjuvant Rx, operation type (High or Low anterior resection) |
| Exp_weighted_sum_errorsminute_no_clash_no_needle | BMI, age, comorbidity, Neoadjuvant Rx, operation type (High or Low anterior resection) |
| Total_Error_number_clash_needle_only | BMI, age, comorbidity, Neoadjuvant Rx, operation type (High or Low anterior resection) |
| Errorsminute_clash_needle_only | BMI, age, comorbidity, Neoadjuvant Rx, operation type (High or Low anterior resection) |
| Linear_weighted_sum_errors_clash_needle_only | BMI, age, comorbidity, Neoadjuvant Rx, operation type (High or Low anterior resection) |
| Linear_weighted_sum_errorsminute_clash_needle_only | BMI, age, comorbidity, Neoadjuvant Rx, operation type (High or Low anterior resection) |
| MGEARS | BMI, age, comorbidity, Neoadjuvant Rx, operation type (High or Low anterior resection) |
| MGEARS_percentage | BMI, age, comorbidity, Neoadjuvant Rx, operation type (High or Low anterior resection) |

Table 5 Number of videos analysed per surgeon and their caseload

| **Surgeon videos analysed and case load** | | | | |
| --- | --- | --- | --- | --- |
|  | Case Frequency | Percent | Robotic case load | Laparoscopic case load |
| Surgeon 1 | 2 | 6.7 | 20.0 | 1000.0 |
| Surgeon 2 | 10 | 33.3 | 800.0 | 200.0 |
| Surgeon 3 | 2 | 6.7 | 300.0 | 1500.0 |
| Surgeon 4 | 3 | 10.0 | Not available | Not available |
| Surgeon 5 | 3 | 10.0 | 400.0 | 300.0 |
| Surgeon 6 | 6 | 20.0 | 60.0 | 200.0 |
| Surgeon 7 | 4 | 13.3 | 10.0 | 2000.0 |
| Total | 30 | 100.0 | N/A | N/A |

Table 6 Descriptive statistics of error group frequencies

| **Descriptive Statistics** | | | | | | | |
| --- | --- | --- | --- | --- | --- | --- | --- |
|  | N | Minimum | Maximum | Sum | Mean | Median | Std. Deviation |
| Total_Error_number | 30 | 9 | 191 | 1540 | 51.33 | 43 | 39.52 |
| Errors/minute | 30 | 0.04 | 0.55 | N/A | 0.24 | 0.20 | 0.15 |
| EAES_1 | 30 | 8 | 156 | 1280 | 42.67 | 37 | 33.60 |
| EAES_2 | 30 | 0 | 35 | 257 | 8.57 | 7 | 6.91 |
| EAES_3 | 30 | 0 | 1 | 3 | 0.10 | 0 | 0.31 |
| EAES_4 | 30 | 0 | 0 | 0 | 0.00 | 0 | 0.00 |
| EAES_5 | 30 | 0 | 0 | 0 | 0.00 | 0 | 0.00 |
| Total_Error_no_clash_no_needle | 30 | 3 | 91 | 538 | 17.93 | 13 | 16.69 |
| Errors/minute_no_clash_no_needle | 30 | 0.02 | 0.26 | N/A | 0.08 | 0.064 | 0.06 |
| EAES_1_no_clash_no_needle | 30 | 0 | 56 | 279 | 9.30 | 6 | 10.68 |
| EAES_2_no_clash_no_needle | 30 | 0 | 35 | 256 | 8.53 | 7 | 6.93 |
| EAES_3_no_clash_no_needle | 30 | 0 | 1 | 3 | 0.10 | 0 | 0.31 |
| EAES_4_no_clash_no_needle | 30 | 0 | 0 | 0 | 0 | 0 | 0 |
| EAES_5_no_clash_no_needle | 30 | 0 | 0 | 0 | 0 | 0 | 0 |
| Total_Error_number_clash_needle_only | 30 | 5 | 100 | 1002 | 33.40 | 26 | 24.80 |
| Errors/minute_clash_needle_only | 30 | 0.02 | 0.36 | N/A | 0.16 | 0.14 | 0.10 |
| EAES_1_clash_needle_only | 30 | 5 | 100 | 1001 | 33.37 | 26 | 24.81 |
| EAES_2_clash_needle_only | 30 | 0 | 1 | 1 | 0.03 | 0 | 0.18 |
| EAES_3_clash_needle_only | 30 | 0 | 0 | 0 | 0 | 0 | 0 |
| EAES_4_clash_needle_only | 30 | 0 | 0 | 0 | 0 | 0 | 0 |
| EAES_5_clash_needle_only | 30 | 0 | 0 | 0 | 0 | 0 | 0 |
| M-GEARS | 30 | 22 | 35 | N/A | 30.97 | 32 | 3.74 |
| M-GEARS_percentage | 30 | 62.86 | 100 | N/A | 88.48 | 91.43 | 10.68 |
| TMEpT_percentage | 24 | 60.42 | 100 | N/A | 75.73 | 82.81 | 25.16 |
| Pre_error_severity_number | 24 | 7 | 109 | 919 | 38.29 | 32.50 | 28.32 |
| Error_severity_number | 24 | 0 | 18 | 196 | 8.17 | 6.50 | 5.21 |
|  |  |  |  |  |  |  |  |

Table 7 Instrument enacting error frequencies

| **Instrument enacting error frequencies** | | |
| --- | --- | --- |
|  | Frequency | Percent |
| Monopolar shears | 264 | 48.4 |
| Grasper - blunt | 164 | 30.1 |
| Laparoscopic blunt grasper | 26 | 4.8 |
| Vessel sealer/synchroseal | 23 | 4.2 |
| Hemoloc/metal clip | 20 | 3.7 |
| Robotic stapler | 18 | 3.3 |
| Other | 9 | 1.7 |
| Camera | 9 | 1.7 |
| Port | 3 | 0.6 |
| Needle holder | 2 | 0.4 |
| Stapler | 2 | 0.4 |
| Suction | 2 | 0.4 |
| Finger | 1 | 0.2 |
| Rectal stapler | 1 | 0.2 |
| Swab | 1 | 0.2 |

Table 8 Recovery mechanism frequencies

| **Recovery mechanism frequencies** | | |
| --- | --- | --- |
|  | Frequency | Percent |
| Corrective action within subtask | 298 | 54.7 |
| Continue uninterrupted | 210 | 32.3 |
| Requires repetition of step | 37 | 6.8 |

Table 9 Correlation between skill and error scores RTME dataset

| **Test** | **Variable** | **Variables** | **Correlation** | **Significance** | **p value** |
| --- | --- | --- | --- | --- | --- |
| Pearsons | MGEARS | Time_operative_videomin | -0.083 | N.S | 0.663 |
| Pearsons | MGEARS | Total_Error_number | -0.639 | Significant | <.001 |
| Pearsons | MGEARS | Error/minute | -0.727 | Significant | <.001 |
| Pearsons | MGEARS | Linear_weighted_sum_errors | -0.651 | Significant | <.001 |
| Pearsons | MGEARS | Linear_weighted_sum_errorsminute | -0.753 | Significant | <.001 |
| Pearsons | MGEARS | Exp_weighted_sum_errors | -0.663 | Significant | <.001 |
| Pearsons | MGEARS | Exp_weighted_sum_errorsminute | -0.772 | Significant | <.001 |
| Pearsons | MGEARS | Total_Error_no_clash_no_needle | -0.538 | Significant | 0.002 |
| Pearsons | MGEARS | Errorsminute_no_clash_no_needle | -0.693 | Significant | <.001 |
| Pearsons | MGEARS | Linear_weighted_sum_errors_no_clash_no_needle | -0.585 | Significant | <.001 |
| Pearsons | MGEARS | Linear_weighted_sum_errorsminute_no_clash_no_needle | -0.749 | Significant | <.001 |
| Pearsons | MGEARS | Exp_weighted_sum_errors_no_clash_no_needle | -0.614 | Significant | <.001 |
| Pearsons | MGEARS | Exp_weighted_sum_errorsminute_no_clash_no_needle | -0.774 | Significant | <.001 |
| Pearsons | MGEARS | Total_Error_number_clash_needle_only | -0.657 | Significant | <.001 |
| Pearsons | MGEARS | Errorsminute_clash_needle_only | -0.689 | Significant | <.001 |
| Pearsons | MGEARS | Linear_weighted_sum_errors_clash_needle_only | -0.654 | Significant | <.001 |
| Pearsons | MGEARS | Linear_weighted_sum_errorsminute_clash_needle_only | -0.683 | Significant | <.001 |
| Spearmans | MGEARS | TMEpt | 0.735 | Significant | 0.007 |
| Pearsons | EAES_1 | EAES_2 | 0.82 | Significant | <.001 |
| Pearsons | EAES_1 | EAES_3 | 0.209 | N.S | 0.269 |
| Spearmans | EAES_1 | EAES_3 | 0.206 | N.S | 0.276 |
| Pearsons | EAES_2 | EAES_3 | 0.021 | N.S | 0.911 |
| Spearmans | EAES_2 | EAES_3 | 0.103 | N.S | 0.588 |
| Pearsons | EAES_1_no_clash_no_needle | EAES_2_no_clash_no_needle | 0.777 | Significant | <0.001 |
| Pearsons | EAES_1_no_clash_no_needle | EAES_3_no_clash_no_needle | 0.16 | N.S | 0.399 |
| Spearmans | EAES_1_no_clash_no_needle | EAES_3_no_clash_no_needle | 0.232 | N.S | 0.217 |
| Pearsons | EAES_2_no_clash_no_needle | EAES_3_no_clash_no_needle | 0.023 | N.S | 0.905 |
| Spearmans | EAES_2_no_clash_no_needle | EAES_3_no_clash_no_needle | 0.11 | N.S | 0.564 |
| Pearsons | EAES_1_clash_needle_only | EAES_2_no_clash_no_needle | 0.776 | Significant | <.001 |
| Pearsons | EAES_1_clash_needle_only | EAES_3_no_clash_no_needle | 0.214 | N.S. | 0.257 |
| Pearsons | EAES_1_clash_needle_only | Error_severity_number | 0.775 | Significant | <0.001 |
| Pearsons | Pre_error_severity_number | Error_severity_number | 0.709 | Significant | <0.001 |

Table 10 VAMIS RTME Summary of significant, and approaching significance, regression models

| **Model** | **DEPENDENT VARIABLE** | **PREDICTOR** | **CONFOUNDERS** | **RESULT** | p value |
| --- | --- | --- | --- | --- | --- |
| Linear | Time_of_operative_video_(minutes)_minus_the_extracorporea_phase | Total_Error_number | None | Approaching significance | 0.054 |
| Linear | Time_of_operative_video_(minutes)_minus_the_extracorporea_phase | Linear_weighted_sum_errors | None | Significant | 0.041 |
| Linear | Time_of_operative_video_(minutes)_minus_the_extracorporea_phase | Exp_weighted_sum_errors | None | Significant | 0.037 |
| Linear | Time_of_operative_video_(minutes)_minus_the_extracorporea_phase | Total_Error_no_clash_no_needle | None | Significant | 0.015 |
| Linear | Time_of_operative_video_(minutes)_minus_the_extracorporea_phase | Linear_weighted_sum_errors_no_clash_no_needle | None | Significant | 0.012 |
| Linear | Time_of_operative_video_(minutes)_minus_the_extracorporea_phase | Exp_weighted_sum_errors_no_clash_no_needle | None | Significant | 0.013 |
| Linear | Time_of_operative_video_(minutes)_minus_the_extracorporea_phase | Shared operating with trainee | None | Approaching significance | 0.079 |
| Linear | Time_of_operative_video_(minutes)_minus_the_extracorporea_phase | Errorsminute_clash_needle_only | bmi, age, comorbidity binary, neoadjuvant binary, ASA, Operation_name_ordinal, Complexity, non-white_ethnicity_binary | Significant, neoadjuvant and operation sig | 0.025 |
| Linear | Time_of_operative_video_(minutes)_minus_the_extracorporea_phase | Linear_weighted_sum_errorsminute_clash_needle_only | bmi, age, comorbidity binary, neoadjuvant binary, ASA, Operation_name_ordinal, Complexity, non-white_ethnicity_binary | Significant, neoadjuvant and operation sig | 0.024 |
| Linear | Time_of_operative_video_(minutes)_minus_the_extracorporea_phase | Shared operating with trainee | bmi, age, comorbidity binary, neoadjuvant binary, ASA, Operation_name_ordinal, Complexity, non-white_ethnicity_binary | Approaching significance, neoadj sig and operation name binary sig | 0.063 |
| Linear | Time_of_operative_video_(minutes)_minus_the_extracorporea_phase | Error | None | Significant | 0.038 |
| Linear | Time_of_operative_video_(minutes)_minus_the_extracorporea_phase | EAES_2 | None | Significant | 0.011 |
| Linear | Time_of_operative_video_(minutes)_minus_the_extracorporea_phase | EAES_1_no_clash_no_needle | None | Significant | 0.033 |
| Linear | Time_of_operative_video_(minutes)_minus_the_extracorporea_phase | EAES_2_no_clash_no_needle | None | Significant | 0.01 |
| Linear | LOS | Total_Error_number | None | Significant | 0.013 |
| Linear | LOS | Error/minute | None | Significant | 0.019 |
| Linear | LOS | Linear_weighted_sum_errors | None | Significant | 0.019 |
| Linear | LOS | Linear_weighted_sum_errorsminute | None | Significant | 0.03 |
| Linear | LOS | Exp_weighted_sum_errors | None | Significant | 0.022 |
| Linear | LOS | Exp_weighted_sum_errorsminute | None | Significant | 0.042 |
| Linear | LOS | Total_Error_no_clash_no_needle | None | Significant | 0.05 |
| Linear | LOS | Errorsminute_no_clash_no_needle | None | Significant | 0.047 |
| Linear | LOS | Linear_weighted_sum_errors_no_clash_no_needle | None | Approaching significance | 0.064 |
| Linear | LOS | Exp_weighted_sum_errors_no_clash_no_needle | None | Approaching significance | 0.068 |
| Linear | LOS | Total_Error_number_clash_needle_only | None | Significant | 0.009 |
| Linear | LOS | Errorsminute_clash_needle_only | None | Significant | 0.018 |
| Linear | LOS | Linear_weighted_sum_errors_clash_needle_only | None | Significant | 0.009 |
| Linear | LOS | Linear_weighted_sum_errorsminute_clash_needle_only | None | Significant | 0.018 |
| Linear | LOS | Pre-error | None | Significant | 0.016 |
| Linear | LOS | EAES_1 | None | Significant | 0.009 |
| Linear | LOS | EAES_1_no_clash_no_needle | None | Significant | 0.034 |
| Linear | LOS | EAES_1_clash_needle_only | None | Significant | 0.009 |
| Linear | LOS | EAES_3 | bmi, age, comorbidity binary, neoadjuvant binary, ASA, Operation_name_ordinal, Complexity, non-white_ethnicity_binary | Approaching significance | 0.055 |
| Linear | LOS | EAES_3_no_clash_no_needle | bmi, age, comorbidity binary, neoadjuvant binary, ASA, Operation_name_ordinal, Complexity, non-white_ethnicity_binary | Approaching significance, operation name | 0.055 |
| Ordinal | Same_admission_HIGHEST_Clavien_Dindo_classification_ordinal | Total_Error_no_clash_no_needle | None | Approaching significance | 0.078 |
| Ordinal | Same_admission_HIGHEST_Clavien_Dindo_classification_ordinal | Total_Error_number | bmi, age, comorbidity binary, neoadjuvant binary, ASA, Operation_name_ordinal, Complexity, non-white_ethnicity_binary | Approaching significance | 0.082 |
| Ordinal | Same_admission_HIGHEST_Clavien_Dindo_classification_ordinal | Error/minute | bmi, age, comorbidity binary, neoadjuvant binary, ASA, Operation_name_ordinal, Complexity, non-white_ethnicity_binary | Approaching significance | 0.08 |
| Ordinal | Same_admission_HIGHEST_Clavien_Dindo_classification_ordinal | Linear_weighted_sum_errors | bmi, age, comorbidity binary, neoadjuvant binary, ASA, Operation_name_ordinal, Complexity, non-white_ethnicity_binary | Approaching significance | 0.061 |
| Ordinal | Same_admission_HIGHEST_Clavien_Dindo_classification_ordinal | Linear_weighted_sum_errorsminute | bmi, age, comorbidity binary, neoadjuvant binary, ASA, Operation_name_ordinal, Complexity, non-white_ethnicity_binary | Approaching significance | 0.071 |
| Ordinal | Same_admission_HIGHEST_Clavien_Dindo_classification_ordinal | Exp_weighted_sum_errors | bmi, age, comorbidity binary, neoadjuvant binary, ASA, Operation_name_ordinal, Complexity, non-white_ethnicity_binary | Approaching significance | 0.081 |
| Ordinal | Same_admission_HIGHEST_Clavien_Dindo_classification_ordinal | Exp_weighted_sum_errorsminute | bmi, age, comorbidity binary, neoadjuvant binary, ASA, Operation_name_ordinal, Complexity, non-white_ethnicity_binary | Approaching significance | 0.067 |
| Ordinal | Same_admission_HIGHEST_Clavien_Dindo_classification_ordinal | Total_Error_no_clash_no_needle | bmi, age, comorbidity binary, neoadjuvant binary, ASA, Operation_name_ordinal, Complexity, non-white_ethnicity_binary | Approaching significance | 0.072 |
| Ordinal | Same_admission_HIGHEST_Clavien_Dindo_classification_ordinal | Errorsminute_no_clash_no_needle | bmi, age, comorbidity binary, neoadjuvant binary, ASA, Operation_name_ordinal, Complexity, non-white_ethnicity_binary | Approaching significance | 0.093 |
| Ordinal | Same_admission_HIGHEST_Clavien_Dindo_classification_ordinal | Linear_weighted_sum_errorsminute_no_clash_no_needle | bmi, age, comorbidity binary, neoadjuvant binary, ASA, Operation_name_ordinal, Complexity, non-white_ethnicity_binary | Approaching significance | 0.094 |
| Ordinal | Same_admission_HIGHEST_Clavien_Dindo_classification_ordinal | Exp_weighted_sum_errors_no_clash_no_needle | bmi, age, comorbidity binary, neoadjuvant binary, ASA, Operation_name_ordinal, Complexity, non-white_ethnicity_binary | Approaching significance | 0.074 |
| Ordinal | Same_admission_HIGHEST_Clavien_Dindo_classification_ordinal | Exp_weighted_sum_errorsminute_no_clash_no_needle | bmi, age, comorbidity binary, neoadjuvant binary, ASA, Operation_name_ordinal, Complexity, non-white_ethnicity_binary | Approaching significance | 0.096 |
| Ordinal | Same_admission_HIGHEST_Clavien_Dindo_classification_ordinal | Errorsminute_clash_needle_only | bmi, age, comorbidity binary, neoadjuvant binary, ASA, Operation_name_ordinal, Complexity, non-white_ethnicity_binary | Approaching significance | 0.087 |
| Ordinal | Same_admission_HIGHEST_Clavien_Dindo_classification_ordinal | Linear_weighted_sum_errorsminute_clash_needle_only | bmi, age, comorbidity binary, neoadjuvant binary, ASA, Operation_name_ordinal, Complexity, non-white_ethnicity_binary | Approaching significance | 0.083 |
| Ordinal | Same_admission_HIGHEST_Clavien_Dindo_classification_ordinal | EAES_1_no_clash_no_needle | None | Approaching significance | 0.051 |
| Ordinal | Same_admission_HIGHEST_Clavien_Dindo_classification_ordinal | EAES_1_no_clash_no_needle | Confounders | Approaching significance | 0.068 |
| Ordinal | Readmission_highest_Clavien_Dindo_classification_ordinal | Errorsminute_no_clash_no_needle | bmi, age, comorbidity binary, neoadjuvant binary, ASA, Operation_name_ordinal, Complexity, non-white_ethnicity_binary | Approaching significance | 0.093 |
| Ordinal | Any_Clavien_Dindo_complication_ordinal | EAES_3 | None | Significant | 0.041 |
| Ordinal | Any_Clavien_Dindo_complication_ordinal | EAES_3_no_clash_no_needle | None | Significant | 0.041 |
| Ordinal | Any_Clavien_Dindo_complication_ordinal | EAES_3 | bmi, age, comorbidity binary, neoadjuvant binary, ASA, Operation_name_ordinal, Complexity, non-white_ethnicity_binary | Significant | 0.031 |
| Ordinal | Any_Clavien_Dindo_complication_ordinal | EAES_3_no_clash_no_needle | bmi, age, comorbidity binary, neoadjuvant binary, ASA, Operation_name_ordinal, Complexity, non-white_ethnicity_binary | Significant | 0.031 |

Table 11 VAMIS RTME Regression models with statistically significant results when adjusting for confounders

| **Coefficients^a^** | | | | | | | | | | |
| --- | --- | --- | --- | --- | --- | --- | --- | --- | --- | --- |
| Model | | Unstandardized Coefficients | | Standardized Coefficients | t | Sig. | 95.0% Confidence Interval for B | | Collinearity Statistics | |
|  |  | B | Std. Error | Beta |  |  | Lower Bound | Upper Bound | Tolerance | VIF |
| 1 | (Constant) | 5.765 | 97.101 |  | 0.059 | 0.953 | -198.236 | 209.766 |  |  |
|  | Errors/minute_clash_needle_only | -345.302 | 141.018 | -0.398 | -2.449 | 0.025 | -641.570 | -49.033 | 0.756 | 1.323 |
|  | BMI | 2.372 | 2.493 | 0.148 | 0.951 | 0.354 | -2.865 | 7.608 | 0.821 | 1.218 |
|  | Age | 0.843 | 0.799 | 0.160 | 1.055 | 0.306 | -0.836 | 2.521 | 0.871 | 1.148 |
|  | Co_morbidities_list_binary | 38.018 | 27.788 | 0.219 | 1.368 | 0.188 | -20.363 | 96.399 | 0.778 | 1.285 |
|  | Neoadjuvant_therapy_Binary | -89.141 | 33.215 | -0.384 | -2.684 | 0.015 | -158.922 | -19.360 | 0.973 | 1.028 |
|  | Operation_name_binary | 98.677 | 25.192 | 0.576 | 3.917 | 0.001 | 45.750 | 151.604 | 0.922 | 1.084 |
| a. Dependent Variable: Time_of_operative_video_(minutes)_minus_the_extracorporea_phase | | | | | | | | | | |
| **Coefficients^a^** | | | | | | | | | | |
| Model | | Unstandardized Coefficients | | Standardized Coefficients | t | Sig. | 95.0% Confidence Interval for B | | Collinearity Statistics | |
|  |  | B | Std. Error | Beta |  |  | Lower Bound | Upper Bound | Tolerance | VIF |
| 1 | (Constant) | 5.608 | 96.756 |  | 0.058 | 0.954 | -197.669 | 208.885 |  |  |
|  | Weighted_sum_errors/minute_clash_needle_only | -1733.309 | 701.339 | -0.399 | -2.471 | 0.024 | -3206.768 | -259.850 | 0.761 | 1.314 |
|  | BMI | 2.409 | 2.478 | 0.151 | 0.972 | 0.344 | -2.796 | 7.615 | 0.827 | 1.209 |
|  | Age | 0.831 | 0.797 | 0.157 | 1.043 | 0.311 | -0.844 | 2.506 | 0.871 | 1.148 |
|  | Co_morbidities_list_binary | 37.639 | 27.659 | 0.217 | 1.361 | 0.190 | -20.470 | 95.748 | 0.782 | 1.279 |
|  | Neoadjuvant_therapy_Binary | -88.317 | 33.130 | -0.381 | -2.666 | 0.016 | -157.920 | -18.715 | 0.973 | 1.028 |
|  | Operation_name_binary | 98.975 | 25.152 | 0.578 | 3.935 | 0.001 | 46.132 | 151.817 | 0.921 | 1.086 |
| a. Dependent Variable: Time_of_operative_video_(minutes)_minus_the_extracorporea_phase | | | | | | | | | | |
| **Coefficients^a^** | | | | | | | | | | |
| Model | | Unstandardized Coefficients | | Standardized Coefficients | t | Sig. | 95.0% Confidence Interval for B | | Collinearity Statistics | |
|  |  | B | Std. Error | Beta |  |  | Lower Bound | Upper Bound | Tolerance | VIF |
| 1 | (Constant) | -21.853 | 99.120 |  | -0.220 | 0.828 | -230.097 | 186.391 |  |  |
|  | Shared operating with trainee | -54.403 | 27.419 | -0.313 | -1.984 | 0.063 | -112.008 | 3.202 | 0.874 | 1.144 |
|  | BMI | 3.224 | 2.520 | 0.201 | 1.280 | 0.217 | -2.069 | 8.517 | 0.879 | 1.137 |
|  | Age | 0.707 | 0.837 | 0.134 | 0.844 | 0.410 | -1.052 | 2.466 | 0.868 | 1.152 |
|  | Co_morbidities_list_binary | 17.151 | 27.646 | 0.099 | 0.620 | 0.543 | -40.931 | 75.233 | 0.860 | 1.163 |
|  | Neoadjuvant_therapy_Binary | -82.370 | 34.801 | -0.355 | -2.367 | 0.029 | -155.484 | -9.256 | 0.969 | 1.032 |
|  | Operation_name_binary | 91.194 | 25.871 | 0.532 | 3.525 | 0.002 | 36.842 | 145.547 | 0.956 | 1.046 |
| a. Dependent Variable: Time_of_operative_video_(minutes)_minus_the_extracorporea_phase | | | | | | | | | | |
| **Coefficients^a^** | | | | | | | | | | |
| Model | | Unstandardized Coefficients | | Standardized Coefficients | t | Sig. | 95.0% Confidence Interval for B | | Collinearity Statistics | |
|  |  | B | Std. Error | Beta |  |  | Lower Bound | Upper Bound | Tolerance | VIF |
| 1 | (Constant) | -0.859 | 7.887 |  | -0.109 | 0.914 | -17.428 | 15.710 |  |  |
|  | EAES_3 | 6.683 | 3.261 | 0.356 | 2.050 | 0.055 | -0.167 | 13.533 | 0.927 | 1.079 |
|  | BMI | -0.375 | 0.195 | -0.326 | -1.920 | 0.071 | -0.785 | 0.035 | 0.965 | 1.036 |
|  | Co_morbidities_list_binary | 3.883 | 2.246 | 0.311 | 1.729 | 0.101 | -0.835 | 8.602 | 0.859 | 1.164 |
|  | Neoadjuvant_therapy_Binary | 1.039 | 2.865 | 0.062 | 0.363 | 0.721 | -4.980 | 7.059 | 0.943 | 1.061 |
|  | Operation_name_binary | 5.625 | 2.086 | 0.457 | 2.696 | 0.015 | 1.242 | 10.007 | 0.970 | 1.031 |
|  | Age | 0.084 | 0.069 | 0.223 | 1.231 | 0.234 | -0.060 | 0.228 | 0.853 | 1.172 |
| a. Dependent Variable: Length of stay (days) | | | | | | | | | | |
| **Parameter Estimates** | | | | | | | | |  |  |
|  | | Estimate | Std. Error | Wald | df | Sig. | 95% Confidence Interval | |  |  |
|  |  |  |  |  |  |  | Lower Bound | Upper Bound |  |  |
| Threshold | [Same_admission_HIGHEST_Clavien_Dindo_classification_ordinal = 0] | 50.595 | 30.280 | 2.792 | 1 | 0.095 | -8.752 | 109.941 |  |  |
|  | [Same_admission_HIGHEST_Clavien_Dindo_classification_ordinal = 2] | 54.020 | 31.240 | 2.990 | 1 | 0.084 | -7.209 | 115.248 |  |  |
| Location | Total_Error_number | 0.081 | 0.046 | 3.023 | 1 | 0.082 | -0.010 | 0.172 |  |  |
|  | BMI | -1.136 | 0.671 | 2.864 | 1 | 0.091 | -2.451 | 0.180 |  |  |
|  | Co_morbidities_list_binary | -3.625 | 4.586 | 0.625 | 1 | 0.429 | -12.612 | 5.363 |  |  |
|  | Neoadjuvant_therapy_Binary | -4.779 | 15.829 | 0.091 | 1 | 0.763 | -35.803 | 26.245 |  |  |
|  | Operation_name_binary | 19.895 | 12.647 | 2.475 | 1 | 0.116 | -4.893 | 44.684 |  |  |
|  | Age | 0.432 | 0.247 | 3.055 | 1 | 0.081 | -0.052 | 0.916 |  |  |
| Link function: Logit. | | | | | | | | |  |  |
| **Parameter Estimates** | | | | | | | | |  |  |
|  | | Estimate | Std. Error | Wald | df | Sig. | 95% Confidence Interval | |  |  |
|  |  |  |  |  |  |  | Lower Bound | Upper Bound |  |  |
| Threshold | [Same_admission_HIGHEST_Clavien_Dindo_classification_ordinal = 0] | 49.129 | 25.392 | 3.743 | 1 | 0.053 | -0.639 | 98.897 |  |  |
|  | [Same_admission_HIGHEST_Clavien_Dindo_classification_ordinal = 2] | 54.772 | 27.882 | 3.859 | 1 | 0.049 | 0.124 | 109.420 |  |  |
| Location | Errorsminute | 36.814 | 21.014 | 3.069 | 1 | 0.080 | -4.374 | 78.001 |  |  |
|  | BMI | -0.515 | 0.398 | 1.675 | 1 | 0.196 | -1.295 | 0.265 |  |  |
|  | Co_morbidities_list_binary | -1.079 | 5.587 | 0.037 | 1 | 0.847 | -12.028 | 9.871 |  |  |
|  | Neoadjuvant_therapy_Binary | -9.957 | 10.212 | 0.951 | 1 | 0.330 | -29.971 | 10.058 |  |  |
|  | Operation_name_binary | 11.048 | 7.669 | 2.075 | 1 | 0.150 | -3.983 | 26.079 |  |  |
|  | Age | 0.365 | 0.195 | 3.509 | 1 | 0.061 | -0.017 | 0.747 |  |  |
| Link function: Logit. | | | | | | | | |  |  |
| **Parameter Estimates** | | | | | | | | |  |  |
|  | | Estimate | Std. Error | Wald | df | Sig. | 95% Confidence Interval | |  |  |
|  |  |  |  |  |  |  | Lower Bound | Upper Bound |  |  |
| Threshold | [Same_admission_HIGHEST_Clavien_Dindo_classification_ordinal = 0] | 67.576 | 36.698 | 3.391 | 1 | 0.066 | -4.350 | 139.502 |  |  |
|  | [Same_admission_HIGHEST_Clavien_Dindo_classification_ordinal = 2] | 71.666 | 37.891 | 3.577 | 1 | 0.059 | -2.600 | 145.931 |  |  |
| Location | Linear_Weighted_sum_errors | 0.433 | 0.231 | 3.513 | 1 | 0.061 | -0.020 | 0.885 |  |  |
|  | BMI | -1.595 | 0.854 | 3.484 | 1 | 0.062 | -3.269 | 0.080 |  |  |
|  | Co_morbidities_list_binary | -5.034 | 5.363 | 0.881 | 1 | 0.348 | -15.546 | 5.478 |  |  |
|  | Neoadjuvant_therapy_Binary | -5.093 | 22.532 | 0.051 | 1 | 0.821 | -49.256 | 39.069 |  |  |
|  | Operation_name_binary | 28.055 | 15.839 | 3.137 | 1 | 0.077 | -2.990 | 59.100 |  |  |
|  | Age | 0.577 | 0.303 | 3.637 | 1 | 0.057 | -0.016 | 1.170 |  |  |
| Link function: Logit. | | | | | | | | |  |  |
| **Parameter Estimates** | | | | | | | | |  |  |
|  | | Estimate | Std. Error | Wald | df | Sig. | 95% Confidence Interval | |  |  |
|  |  |  |  |  |  |  | Lower Bound | Upper Bound |  |  |
| Threshold | [Same_admission_HIGHEST_Clavien_Dindo_classification_ordinal = 0] | 47.549 | 24.325 | 3.821 | 1 | 0.051 | -0.127 | 95.224 |  |  |
|  | [Same_admission_HIGHEST_Clavien_Dindo_classification_ordinal = 2] | 52.789 | 26.483 | 3.973 | 1 | 0.046 | 0.884 | 104.694 |  |  |
| Location | Linear_Weighted_sum_errorsminute | 145.976 | 80.988 | 3.249 | 1 | 0.071 | -12.758 | 304.710 |  |  |
|  | BMI | -0.605 | 0.413 | 2.146 | 1 | 0.143 | -1.415 | 0.205 |  |  |
|  | Co_morbidities_list_binary | -1.749 | 5.112 | 0.117 | 1 | 0.732 | -11.768 | 8.269 |  |  |
|  | Neoadjuvant_therapy_Binary | -9.451 | 8.878 | 1.133 | 1 | 0.287 | -26.852 | 7.949 |  |  |
|  | Operation_name_binary | 12.462 | 7.974 | 2.442 | 1 | 0.118 | -3.167 | 28.092 |  |  |
|  | Age | 0.358 | 0.188 | 3.631 | 1 | 0.057 | -0.010 | 0.725 |  |  |
| Link function: Logit. | | | | | | | | |  |  |
| **Parameter Estimates** | | | | | | | | |  |  |
|  | | Estimate | Std. Error | Wald | df | Sig. | 95% Confidence Interval | |  |  |
|  |  |  |  |  |  |  | Lower Bound | Upper Bound |  |  |
| Threshold | [Same_admission_HIGHEST_Clavien_Dindo_classification_ordinal = 0] | 106.796 | 60.269 | 3.140 | 1 | 0.076 | -11.330 | 224.922 |  |  |
|  | [Same_admission_HIGHEST_Clavien_Dindo_classification_ordinal = 2] | 113.358 | 62.641 | 3.275 | 1 | 0.070 | -9.416 | 236.132 |  |  |
| Location | Exp_Weighted_sum_errors | 6.681 | 3.832 | 3.039 | 1 | 0.081 | -0.830 | 14.193 |  |  |
|  | BMI | -2.562 | 1.426 | 3.226 | 1 | 0.072 | -5.357 | 0.234 |  |  |
|  | Co_morbidities_list_binary | -8.155 | 8.908 | 0.838 | 1 | 0.360 | -25.614 | 9.305 |  |  |
|  | Neoadjuvant_therapy_Binary | -4.986 | 34.556 | 0.021 | 1 | 0.885 | -72.714 | 62.743 |  |  |
|  | Operation_name_binary | 45.197 | 26.153 | 2.987 | 1 | 0.084 | -6.063 | 96.457 |  |  |
|  | Age | 0.913 | 0.501 | 3.320 | 1 | 0.068 | -0.069 | 1.894 |  |  |
| Link function: Logit. | | | | | | | | |  |  |
| **Parameter Estimates** | | | | | | | | |  |  |
|  | | Estimate | Std. Error | Wald | df | Sig. | 95% Confidence Interval | |  |  |
|  |  |  |  |  |  |  | Lower Bound | Upper Bound |  |  |
| Threshold | [Same_admission_HIGHEST_Clavien_Dindo_classification_ordinal = 0] | 48.073 | 24.458 | 3.863 | 1 | 0.049 | 0.136 | 96.009 |  |  |
|  | [Same_admission_HIGHEST_Clavien_Dindo_classification_ordinal = 2] | 53.114 | 26.432 | 4.038 | 1 | 0.044 | 1.308 | 104.920 |  |  |
| Location | Exp_Weighted_sum_errorsminute | 1398.193 | 763.395 | 3.355 | 1 | 0.067 | -98.033 | 2894.419 |  |  |
|  | BMI | -0.647 | 0.422 | 2.352 | 1 | 0.125 | -1.475 | 0.180 |  |  |
|  | Co_morbidities_list_binary | -1.720 | 5.484 | 0.098 | 1 | 0.754 | -12.468 | 9.029 |  |  |
|  | Neoadjuvant_therapy_Binary | -9.198 | 8.435 | 1.189 | 1 | 0.276 | -25.731 | 7.335 |  |  |
|  | Operation_name_binary | 13.250 | 8.211 | 2.604 | 1 | 0.107 | -2.843 | 29.343 |  |  |
|  | Age | 0.363 | 0.188 | 3.719 | 1 | 0.054 | -0.006 | 0.731 |  |  |
| Link function: Logit. | | | | | | | | |  |  |
| **Parameter Estimates** | | | | | | | | |  |  |
|  | | Estimate | Std. Error | Wald | df | Sig. | 95% Confidence Interval | |  |  |
|  |  |  |  |  |  |  | Lower Bound | Upper Bound |  |  |
| Threshold | [Same_admission_HIGHEST_Clavien_Dindo_classification_ordinal = 0] | 48.732 | 29.653 | 2.701 | 1 | 0.100 | -9.388 | 106.851 |  |  |
|  | [Same_admission_HIGHEST_Clavien_Dindo_classification_ordinal = 2] | 52.525 | 30.660 | 2.935 | 1 | 0.087 | -7.568 | 112.618 |  |  |
| Location | Total_Error_no_clash_no_needle | 0.139 | 0.077 | 3.247 | 1 | 0.072 | -0.012 | 0.289 |  |  |
|  | BMI | -1.286 | 0.744 | 2.991 | 1 | 0.084 | -2.744 | 0.171 |  |  |
|  | Co_morbidities_list_binary | -2.282 | 3.970 | 0.330 | 1 | 0.565 | -10.064 | 5.499 |  |  |
|  | Neoadjuvant_therapy_Binary | -5.261 | 13.683 | 0.148 | 1 | 0.701 | -32.078 | 21.557 |  |  |
|  | Operation_name_binary | 21.509 | 13.466 | 2.551 | 1 | 0.110 | -4.885 | 47.903 |  |  |
|  | Age | 0.443 | 0.252 | 3.081 | 1 | 0.079 | -0.052 | 0.938 |  |  |
| Link function: Logit. | | | | | | | | |  |  |
| **Parameter Estimates** | | | | | | | | |  |  |
|  | | Estimate | Std. Error | Wald | df | Sig. | 95% Confidence Interval | |  |  |
|  |  |  |  |  |  |  | Lower Bound | Upper Bound |  |  |
| Threshold | [Same_admission_HIGHEST_Clavien_Dindo_classification_ordinal = 0] | 60.389 | 35.845 | 2.838 | 1 | 0.092 | -9.865 | 130.643 |  |  |
|  | [Same_admission_HIGHEST_Clavien_Dindo_classification_ordinal = 2] | 66.614 | 37.691 | 3.124 | 1 | 0.077 | -7.258 | 140.486 |  |  |
| Location | Errorsminute_no_clash_no_needle | 69.826 | 41.570 | 2.821 | 1 | 0.093 | -11.651 | 151.302 |  |  |
|  | BMI | -1.324 | 0.789 | 2.818 | 1 | 0.093 | -2.870 | 0.222 |  |  |
|  | Co_morbidities_list_binary | -2.499 | 5.432 | 0.212 | 1 | 0.645 | -13.145 | 8.147 |  |  |
|  | Neoadjuvant_therapy_Binary | -10.174 | 14.518 | 0.491 | 1 | 0.483 | -38.629 | 18.281 |  |  |
|  | Operation_name_binary | 23.282 | 14.795 | 2.476 | 1 | 0.116 | -5.715 | 52.279 |  |  |
|  | Age | 0.522 | 0.296 | 3.106 | 1 | 0.078 | -0.058 | 1.103 |  |  |
| Link function: Logit. | | | | | | | | |  |  |
| **Parameter Estimates** | | | | | | | | |  |  |
|  | | Estimate | Std. Error | Wald | df | Sig. | 95% Confidence Interval | |  |  |
|  |  |  |  |  |  |  | Lower Bound | Upper Bound |  |  |
| Threshold | [Same_admission_HIGHEST_Clavien_Dindo_classification_ordinal = 0] | 62.330 | 36.489 | 2.918 | 1 | 0.088 | -9.186 | 133.847 |  |  |
|  | [Same_admission_HIGHEST_Clavien_Dindo_classification_ordinal = 2] | 68.380 | 38.411 | 3.169 | 1 | 0.075 | -6.904 | 143.665 |  |  |
| Location | Linear_Weighted_sum_errorsminute_no_clash_no_needle | 274.008 | 163.542 | 2.807 | 1 | 0.094 | -46.529 | 594.545 |  |  |
|  | BMI | -1.392 | 0.820 | 2.878 | 1 | 0.090 | -3.000 | 0.216 |  |  |
|  | Co_morbidities_list_binary | -4.225 | 5.767 | 0.537 | 1 | 0.464 | -15.528 | 7.078 |  |  |
|  | Neoadjuvant_therapy_Binary | -12.120 | 13.736 | 0.778 | 1 | 0.378 | -39.043 | 14.803 |  |  |
|  | Operation_name_binary | 25.028 | 15.426 | 2.632 | 1 | 0.105 | -5.207 | 55.263 |  |  |
|  | Age | 0.526 | 0.298 | 3.120 | 1 | 0.077 | -0.058 | 1.109 |  |  |
| Link function: Logit. | | | | | | | | |  |  |
| **Parameter Estimates** | | | | | | | | |  |  |
|  | | Estimate | Std. Error | Wald | df | Sig. | 95% Confidence Interval | |  |  |
|  |  |  |  |  |  |  | Lower Bound | Upper Bound |  |  |
| Threshold | [Same_admission_HIGHEST_Clavien_Dindo_classification_ordinal = 0] | 49.758 | 30.254 | 2.705 | 1 | 0.100 | -9.540 | 109.055 |  |  |
|  | [Same_admission_HIGHEST_Clavien_Dindo_classification_ordinal = 2] | 53.552 | 31.271 | 2.933 | 1 | 0.087 | -7.738 | 114.841 |  |  |
| Location | Exp_Weighted_sum_errors_no_clash_no_needle | 5.054 | 2.833 | 3.182 | 1 | 0.074 | -0.499 | 10.608 |  |  |
|  | BMI | -1.365 | 0.788 | 3.003 | 1 | 0.083 | -2.910 | 0.179 |  |  |
|  | Co_morbidities_list_binary | -3.626 | 4.289 | 0.715 | 1 | 0.398 | -12.032 | 4.781 |  |  |
|  | Neoadjuvant_therapy_Binary | -4.671 | 14.013 | 0.111 | 1 | 0.739 | -32.136 | 22.795 |  |  |
|  | Operation_name_binary | 22.835 | 14.160 | 2.601 | 1 | 0.107 | -4.917 | 50.587 |  |  |
|  | Age | 0.449 | 0.257 | 3.061 | 1 | 0.080 | -0.054 | 0.953 |  |  |
| Link function: Logit. | | | | | | | | |  |  |
| **Parameter Estimates** | | | | | | | | |  |  |
|  | | Estimate | Std. Error | Wald | df | Sig. | 95% Confidence Interval | |  |  |
|  |  |  |  |  |  |  | Lower Bound | Upper Bound |  |  |
| Threshold | [Same_admission_HIGHEST_Clavien_Dindo_classification_ordinal = 0] | 62.191 | 36.387 | 2.921 | 1 | 0.087 | -9.127 | 133.508 |  |  |
|  | [Same_admission_HIGHEST_Clavien_Dindo_classification_ordinal = 2] | 68.360 | 38.359 | 3.176 | 1 | 0.075 | -6.823 | 143.543 |  |  |
| Location | Exp_Weighted_sum_errorsminute_no_clash_no_needle | 2631.427 | 1582.794 | 2.764 | 1 | 0.096 | -470.793 | 5733.647 |  |  |
|  | BMI | -1.381 | 0.816 | 2.864 | 1 | 0.091 | -2.980 | 0.218 |  |  |
|  | Co_morbidities_list_binary | -5.096 | 6.278 | 0.659 | 1 | 0.417 | -17.399 | 7.208 |  |  |
|  | Neoadjuvant_therapy_Binary | -13.521 | 13.569 | 0.993 | 1 | 0.319 | -40.116 | 13.074 |  |  |
|  | Operation_name_binary | 25.073 | 15.419 | 2.644 | 1 | 0.104 | -5.148 | 55.294 |  |  |
|  | Age | 0.517 | 0.294 | 3.100 | 1 | 0.078 | -0.059 | 1.093 |  |  |
| Link function: Logit. | | | | | | | | |  |  |
| **Parameter Estimates** | | | | | | | | |  |  |
|  | | Estimate | Std. Error | Wald | df | Sig. | 95% Confidence Interval | |  |  |
|  |  |  |  |  |  |  | Lower Bound | Upper Bound |  |  |
| Threshold | [Same_admission_HIGHEST_Clavien_Dindo_classification_ordinal = 0] | 66.766 | 36.552 | 3.336 | 1 | 0.068 | -4.876 | 138.407 |  |  |
|  | [Same_admission_HIGHEST_Clavien_Dindo_classification_ordinal = 2] | 72.011 | 38.152 | 3.563 | 1 | 0.059 | -2.766 | 146.788 |  |  |
| Location | Errorsminute_clash_needle_only | 87.026 | 50.847 | 2.929 | 1 | 0.087 | -12.632 | 186.684 |  |  |
|  | BMI | -0.127 | 0.425 | 0.090 | 1 | 0.764 | -0.959 | 0.705 |  |  |
|  | Co_morbidities_list_binary | 0.678 | 7.348 | 0.009 | 1 | 0.926 | -13.724 | 15.080 |  |  |
|  | Neoadjuvant_therapy_Binary | -13.779 | 11.039 | 1.558 | 1 | 0.212 | -35.415 | 7.857 |  |  |
|  | Operation_name_binary | 8.894 | 7.196 | 1.528 | 1 | 0.216 | -5.210 | 22.999 |  |  |
|  | Age | 0.414 | 0.224 | 3.424 | 1 | 0.064 | -0.024 | 0.852 |  |  |
| Link function: Logit. | | | | | | | | |  |  |
| **Parameter Estimates** | | | | | | | | |  |  |
|  | | Estimate | Std. Error | Wald | df | Sig. | 95% Confidence Interval | |  |  |
|  |  |  |  |  |  |  | Lower Bound | Upper Bound |  |  |
| Threshold | [Same_admission_HIGHEST_Clavien_Dindo_classification_ordinal = 0] | 66.310 | 35.600 | 3.469 | 1 | 0.063 | -3.465 | 136.085 |  |  |
|  | [Same_admission_HIGHEST_Clavien_Dindo_classification_ordinal = 2] | 71.647 | 37.278 | 3.694 | 1 | 0.055 | -1.417 | 144.711 |  |  |
| Location | Weighted_sum_errorsminute_clash_needle_only | 444.820 | 256.506 | 3.007 | 1 | 0.083 | -57.924 | 947.563 |  |  |
|  | BMI | -0.111 | 0.401 | 0.077 | 1 | 0.781 | -0.898 | 0.675 |  |  |
|  | Co_morbidities_list_binary | 0.141 | 6.754 | 0.000 | 1 | 0.983 | -13.096 | 13.379 |  |  |
|  | Neoadjuvant_therapy_Binary | -13.836 | 11.080 | 1.559 | 1 | 0.212 | -35.552 | 7.879 |  |  |
|  | Operation_name_binary | 8.727 | 6.694 | 1.700 | 1 | 0.192 | -4.392 | 21.846 |  |  |
|  | Age | 0.408 | 0.217 | 3.543 | 1 | 0.060 | -0.017 | 0.832 |  |  |
| Link function: Logit. | | | | | | | | |  |  |
| **Parameter Estimates** | | | | | | | | |  |  |
|  | | Estimate | Std. Error | Wald | df | Sig. | 95% Confidence Interval | |  |  |
|  |  |  |  |  |  |  | Lower Bound | Upper Bound |  |  |
| Threshold | [Same_admission_HIGHEST_Clavien_Dindo_classification_ordinal = 0] | 66.410 | 37.542 | 3.129 | 1 | 0.077 | -7.171 | 139.991 |  |  |
|  | [Same_admission_HIGHEST_Clavien_Dindo_classification_ordinal = 2] | 72.068 | 39.254 | 3.371 | 1 | 0.066 | -4.868 | 149.005 |  |  |
| Location | EAES_1_no_clash_no_needle | 0.279 | 0.152 | 3.339 | 1 | 0.068 | -0.020 | 0.577 |  |  |
|  | BMI | -1.684 | 0.916 | 3.379 | 1 | 0.066 | -3.479 | 0.111 |  |  |
|  | Co_morbidities_list_binary | -1.050 | 4.913 | 0.046 | 1 | 0.831 | -10.680 | 8.579 |  |  |
|  | Neoadjuvant_therapy_Binary | -7.904 | 17.868 | 0.196 | 1 | 0.658 | -42.924 | 27.116 |  |  |
|  | Operation_name_binary | 28.024 | 16.311 | 2.952 | 1 | 0.086 | -3.946 | 59.994 |  |  |
|  | Age | 0.613 | 0.327 | 3.515 | 1 | 0.061 | -0.028 | 1.254 |  |  |
| Link function: Logit. | | | | | | | | |  |  |
| **Parameter Estimates** | | | | | | | | |  |  |
|  | | Estimate | Std. Error | Wald | df | Sig. | 95% Confidence Interval | |  |  |
|  |  |  |  |  |  |  | Lower Bound | Upper Bound |  |  |
| Threshold | [Same_admission_HIGHEST_Clavien_Dindo_classification_ordinal = 0]RTM | 60.389 | 35.845 | 2.838 | 1 | 0.092 | -9.865 | 130.643 |  |  |
|  | [Same_admission_HIGHEST_Clavien_Dindo_classification_ordinal = 2] | 66.614 | 37.691 | 3.124 | 1 | 0.077 | -7.258 | 140.486 |  |  |
| Location | Errorsminute_no_clash_no_needle | 69.826 | 41.570 | 2.821 | 1 | 0.093 | -11.651 | 151.302 |  |  |
|  | BMI | -1.324 | 0.789 | 2.818 | 1 | 0.093 | -2.870 | 0.222 |  |  |
|  | Co_morbidities_list_binary | -2.499 | 5.432 | 0.212 | 1 | 0.645 | -13.145 | 8.147 |  |  |
|  | Neoadjuvant_therapy_Binary | -10.174 | 14.518 | 0.491 | 1 | 0.483 | -38.629 | 18.281 |  |  |
|  | Operation_name_binary | 23.282 | 14.795 | 2.476 | 1 | 0.116 | -5.715 | 52.279 |  |  |
|  | Age | 0.522 | 0.296 | 3.106 | 1 | 0.078 | -0.058 | 1.103 |  |  |
| Link function: Logit. | | | | | | | | |  |  |
| **Parameter Estimates** | | | | | | | | |  |  |
|  | | Estimate | Std. Error | Wald | df | Sig. | 95% Confidence Interval | |  |  |
|  |  |  |  |  |  |  | Lower Bound | Upper Bound |  |  |
| Threshold | [Any_Clavien_Dindo_complication_ordinal = .00] | 22.959 | 11.580 | 3.931 | 1 | 0.047 | 0.262 | 45.657 |  |  |
|  | [Any_Clavien_Dindo_complication_ordinal = 2.00] | 25.293 | 12.201 | 4.297 | 1 | 0.038 | 1.379 | 49.207 |  |  |
| Location | EAES_3 | 6.716 | 3.122 | 4.627 | 1 | 0.031 | 0.597 | 12.836 |  |  |
|  | BMI | 0.161 | 0.111 | 2.106 | 1 | 0.147 | -0.056 | 0.378 |  |  |
|  | Co_morbidities_list_binary | 5.826 | 4.192 | 1.932 | 1 | 0.165 | -2.389 | 14.042 |  |  |
|  | Neoadjuvant_therapy_Binary | -19.290 | 0.000 |  | 1 |  | -19.290 | -19.290 |  |  |
|  | Operation_name_binary | 1.642 | 1.457 | 1.270 | 1 | 0.260 | -1.214 | 4.499 |  |  |
|  | Age | 0.129 | 0.091 | 2.002 | 1 | 0.157 | -0.050 | 0.309 |  |  |
| Link function: Logit. | | | | | | | | |  |  |
| **Parameter Estimates** | | | | | | | | |  |  |
|  | | Estimate | Std. Error | Wald | df | Sig. | 95% Confidence Interval | |  |  |
|  |  |  |  |  |  |  | Lower Bound | Upper Bound |  |  |
| Threshold | [Any_Clavien_Dindo_complication_ordinal = .00] | 22.959 | 11.580 | 3.931 | 1 | 0.047 | 0.262 | 45.657 |  |  |
|  | [Any_Clavien_Dindo_complication_ordinal = 2.00] | 25.293 | 12.201 | 4.297 | 1 | 0.038 | 1.379 | 49.207 |  |  |
| Location | EAES_3_no_clash_no_needle | 6.716 | 3.122 | 4.627 | 1 | 0.031 | 0.597 | 12.836 |  |  |
|  | BMI | 0.161 | 0.111 | 2.106 | 1 | 0.147 | -0.056 | 0.378 |  |  |
|  | Co_morbidities_list_binary | 5.826 | 4.192 | 1.932 | 1 | 0.165 | -2.389 | 14.042 |  |  |
|  | Neoadjuvant_therapy_Binary | -19.290 | 0.000 |  | 1 |  | -19.290 | -19.290 |  |  |
|  | Operation_name_binary | 1.642 | 1.457 | 1.270 | 1 | 0.260 | -1.214 | 4.499 |  |  |
|  | Age | 0.129 | 0.091 | 2.002 | 1 | 0.157 | -0.050 | 0.309 |  |  |
| Link function: Logit. | | | | | | | | |  |  |
